# Chemical composition and insecticidal activity of essential oils from *Citrus limon*, *Citrus aurantium*, and *Citrus margarita* against *Musca domestica*

**DOI:** 10.1038/s41598-026-48049-6

**Published:** 2026-04-20

**Authors:** Abeer H. Elmaidomy, Esraa M. Mohamed, Radwa Taher Mohie el-dien, Hesham A. Abou-Zied, Azza A. Mostafa, Riham H. Taha, Mohamed A. El-Badry, Usama Ramadan Abdelmohsen, Khayrya A. Youssif

**Affiliations:** 1 Department of Pharmacognosy, Faculty of Pharmacy, Beni-Suef University, Beni-Suef, 62514 Egypt; 2 Department of Pharmacognosy, Faculty of Pharmacy, MUST, Giza, 12566 Egypt; 3 Department of Pharmacognosy, Faculty of Pharmacy, Deraya University, Minia, 61111 Egypt; 4 Department of Medicinal Chemistry, Faculty of Pharmacy, Deraya University, Minia, 61111 Egypt; 5 Research Institute of Medical Entomology (RIME), General Organization for Teaching Hospitals and Institutes (GOTHI), Giza, Egypt; 6 Department of Pharmacognosy, Faculty of Pharmacy, Minia University, Minia, 61519 Egypt; 7 Department of Pharmacognosy, Faculty of Pharmacy, El Saleheya El Gadida University, El Sharqya, Egypt

**Keywords:** Citrus species, GC/MS analysis, Essential oils, Insecticidal activity, *Musca domestica*, Biochemistry, Chemical biology, Chemistry, Drug discovery, Plant sciences

## Abstract

There are numerous culinary and therapeutic applications for citrus peel essential oils. In this study, we used GC/MS analysis to investigate the differences in yield and chemical composition of the essential oils extracted from the fresh peels of three *Citrus* species: *C. limon*, * C. aurantium*, and *C. margarita*. In total, 21 compounds were isolated from *C. limon*, 15 from *C. aurantium*, and 9 from *C. margarita*. The most prevalent constituents were α-myrcene (ranging from 7.05 to 12.80% and 11.50%) and D-limonene (ranging from 15.46 to 71.71% and 71.59%). Furthermore, the essential oils of the three citrus species were tested for their ability to kill adult houseflies (*Musca domestica*). The results showed that *C. limon* oil was more effective than *C. aurantium* and *C. margarita* oils, with an LC_50_ value of 3.19 and a 100% toxicity index. An in silico study assessed 31 Citrus-derived compounds for their insecticidal potential against *Musca domestica* by targeting acetylcholinesterase (AChE). Network and docking analysis identified geranyl acetate as the top binder (− 10.2 kcal/mol), interacting with key residues involved in neurotransmission. A 100 ns molecular dynamics confirmed its stable binding and supports its potential role as a natural AChE inhibitor for insect control. These findings offer a molecular basis for upcoming experimental verification and advancement of environmentally benign pesticides derived from citrus.

**Supplementary Information:**

The online version contains supplementary material available at 10.1038/s41598-026-48049-6.

## Introduction

One of the most commercially important commodities cultivated globally in terms of both area and production value is the *Citrus* genus, which belongs to the Rutaceae family. This evergreen shrub is believed to be native to Southeast Asia, the Himalayan region of southern China, northeastern India, and northern Burma^1^.

Citrus is currently found in more than 140 countries. Around 144 million tons of citrus were produced worldwide in 2019 on 9.89 million hectares of growing land, according to the Food and Agriculture Organization of the United Nations (FAO). China led the globe in this regard, producing 38 million tons of citrus and cultivating 2.88 million hectares of citrus. The most important citrus species for the commercial sector are *C. sinensis*, *C. aurantium*, *C. reticulata*, *C. paradisi*, *C. grandis*, *C. limon*, *C. medica*, *C. aurantifolia*, *C. japonica*, and hybrids^1^.

The *Citrus* genus has immense industrial value around the world, owing not only to its healthy, juicy, and valuable fruits, but also to the essential oils contained in its numerous vegetative components. As a result, citrus fruits and citrus essential oils can help to improve the nation’s economy and create jobs. Because of their diverse physiologically active terpene group constituents and appealing scents, citrus essential oils have already established significant positions in the culinary, cosmetic, and flavor industries. Additionally, because of their antibacterial, anticancer, antioxidant, anti-inflammatory, and metabolic disorder-relieving qualities, these oils and their components have become more significant in the pharmaceutical and medical sectors during the past few decades^2^.

Citrus essential oils (EOs) have been shown in numerous studies to have insecticidal properties; some of these EOs are sold commercially for consumer use against insect pests. The order Diptera has several insects that carry disease, making them more hazardous to human and animal health than any other group. Among these insects is the housefly, which has a close ecological niche with humans^3^.

The common bug *Musca domestica* L. ^4^ can be found in waste areas such as cattle ranches and trash dumps^5^. Numerous eggs are typically deposited on organic substrates such as refuse and faeces, where they progress through the stages of larvae, pupae, and adults. The duration for an egg to mature into an adult fly varies from 6 to 42 days, contingent upon the temperature. The food consumed also influences the duration of each stage^6^.

It is not merely an irritating annoyance but also a significant mechanical vector for over 100 detrimental species, including parasitic worms and pathogens responsible for diseases such as cholera, typhoid, bacillary dysentery, tuberculosis, anthrax, ophthalmia neonatorum, and infantile diarrhea^7^. It can contaminate human food via mechanical contact with infected external body parts or through the regurgitation and faeces of houseflies during feeding. It is also transmitted through sewage, refuse, and other sources of contamination^8^. Chemical control has been utilised using a range of pesticides to mitigate this problem. The pest control industry has traditionally relied on synthetic insecticides; however, their overuse and indiscriminate application have resulted in food poisoning and environmental deterioration^9^. Chemical pesticides may exhibit mutagenic, cytotoxic, and carcinogenic effects on humans^10^.

Insecticides typically harm organisms other than insects, including humans, due to their non-specific mode of action. Research by the WHO and the United Nations Environment Programme (UNEP) indicates that pesticide exposure results in 200,000 fatalities and three million cases of poisoning worldwide, predominantly in developing countries^11^. Plant essential oils are experiencing a resurgence in popularity due to increasing concerns for eco-friendly products, as they serve as effective pesticides while being relatively safe and environmentally benign compared to synthetic alternatives. Numerous essential oils contribute to plants’ natural defence mechanisms and are utilised in integrated pest and disease management strategies. Some citrus species are believed to contain specific bioactive compounds in their essential oils, which confer resistance to certain insects and diseases^3^.

Regrettably, due to its short lifespan and fast reproduction, the housefly is increasingly developing resistance to insecticides. To utilize citrus components as insecticidal and repellent agents, it is essential to comprehend their interactions with houseflies. Several studies have demonstrated that citrus essential oil exerts beneficial effects on houseflies. The plant is consumable, ensuring safety against residual contamination or toxicity to users, a benefit of considering the CEOs as insecticidal and insect-repellent. Also, the pleasant aroma enhances consumer acceptance.

This study strives to evaluate the insecticidal effectiveness of three *Citrus* species: *C. limon*, *C. aurantium*, and *C. margarita*, alongside the identification of the primary active metabolites in their essential oils using GC/MS analysis. The molecular dynamics study connects insecticidal efficiency with the primary active metabolites in citrus species.

## Materials and methods

### Fruit collection

Fully mature fruits of *Citrus limon*, *C. aurantium*, and *C. margarita* (Kumquat) were harvested from a residential garden on Atia Street, Beni-Suef, Egypt, after obtaining permission from the property owner in January 2023. Dr. Abd El-Halim A. Mohammed from the Department of Flora and Phytotaxonomy Research at the Horticulture Research Institute in Dokki, Cairo, Egypt, kindly provided the identifications. Herbarium specimens were submitted to the Department of Pharmacognosy, Faculty of Pharmacy, Beni-Suef University, Egypt, with reference numbers (2021-BuPD-102, 104, and 114).

### Sample preparation

The fresh peels (0.25 kg) were ground and hydrodistilled separately for two hours at 75 °C using the Clevenger apparatus. The oils were collected individually, dehydrated using anhydrous sodium sulphate, and stored in sealed amber glass vials at 4 °C. The yield (v/w %) was calculated based on the fresh weight of the plant material^12–17^.

### GC-MS analysis

Gas chromatography-mass spectrometry (GC/MS) was used to perform chromatographic analysis on the oil recovered from the peels^12,13,16,18–22^. The GC/MS apparatus combines a thermal mass spectrometer detector (ISQ single quadrupole mass spectrometer) with a TRACE GC ultra-high-performance gas chromatograph (Thermo Scientific Corp., USA). A TR-5 MS column (30 m x 0.32 mm i.d., 0.25 mm film thickness) was installed in the GC/MS system. For the analysis, Helium was used as the carrier gas, and the split ratio was set to 1:10 using the following temperature program: 60 °C for 1 min, then 4.0 °C/min to 240 °C and a 1-minute hold. The injector and detector were maintained at 210 °C. One-liter samples of the mixes were always administered as diluted samples (1:10 hexane, v/v). Mass spectra were produced using a spectral range of m/z 40–450 and electron ionisation (EI) at 70 eV. Using AMDIS software (https://www.amdis.net), the chemical components of the essential oil were deconvoluted and identified by their retention indices (relative to n-alkanes C-8 ~ C-22), mass spectra matching genuine standards, and retention times (when available). (NIST Standard Reference Database, 78 Version 5.10) Wiley spectral library collection^23–25^.

### Test housefly

Twenty-five insects of the laboratory strain of the housefly, *M. domestica*, were obtained from the Research Institute of Medical Entomology, Cairo, Egypt, aged 2–5 days. It was reared in cages provided with saturated cotton by powdered milk dissolved in water at 28–30 ± 1 °C, and 55–60% RH. Following laying eggs, the female housefly was transferred to cotton on an artificial diet consisting of 200 g of wheat bran and 150 ml of distilled water, designed to sustain adult houseflies for subsequent bioassay evaluations.

### Testing the toxicity of *Citrus limon*, *Citrus aurantium*, and *Citrus margarita* peel oils against *Musca domestica* adults

The bioassay tests were conducted on housefly adults at the Research Institute of Medical Entomology according to the CDC bioassay technique^26^. For each material, CDC bottles were coated with a wide range of concentrations and tested in the adult bioassay to assess the lethal impact on adult house flies. After that, record how many insects are dead or alive every 15 min until all are dead or up to 2 h, to estimate the mortality percentage, to calculate LC_50_ and LC_90_ (the concentrations of the materials that produce 50% and 90% mortality, respectively, in the exposed flies after 2 h). Each test was conducted in four independent trials, and each trial was repeated 4 times on different dates for each concentration under laboratory conditions of 27 ± 2 °C and 70 ± 10% RH. Mean mortality data from the four replicates per dose were used to calculate the LC_50_. The control was prepared by coating the CDC bottles with only water. Abbott’s formula^27^ It was recommended to correct the results if the mortality within two hours in the control bottle was between 3% and 10%. Discarding the bioassay results was performed if mortality in the control bottle at the end of the test exceeded 10%. Houseflies were considered dead if they could no longer stand.

### In silico studies

#### Protein–protein interaction network (PPI) analysis

To explore the molecular interactions of bioactive compounds identified from experimental studies of *Musca domestica* and their potential insecticidal activity, key compounds were analyzed using the STRING database. STRING (https://string-db.org/) is a powerful tool for constructing protein–protein interaction (PPI) networks, and was employed to map how these compounds interact with protein targets in *Musca domestica*. The main goal was to identify critical insect-related targets that may play significant roles in resistance mechanisms^28^. A confidence score threshold of 0.4 was applied to filter for high-confidence interactions, ensuring that only the most relevant molecular connections were included in the analysis. Network visualization and interpretation were performed using Cytoscape (https://cytoscape.org/), a robust tool for biological network analysis^29^.

#### Gene ontology and pathway enrichment analysis

To gain deeper insights into the biological roles and molecular interactions of the insecticide resistance-related genes in *Musca domestica*, Gene Ontology (GO) and Kyoto Encyclopedia of Genes and Genomes (KEGG) pathway enrichment analyses were performed^30^. First, the related insecticide resistance genes were retrieved from GenBank (https://www.ncbi.nlm.nih.gov/genbank), a comprehensive repository of publicly available genetic sequences. The genes from *Musca domestica* were specifically selected for analysis, as they play a critical role in resistance to inhibitors. After retrieving the gene sequences, their corresponding gene products (the proteins they encode) were obtained. These proteins are pivotal in understanding how the genes function at the molecular level, particularly in relation to resistance mechanisms to insecticides. Next, the STITCH database (http://stitch.embl.de) was used to correlate the major bioactive compounds identified in the essential oils with these insecticide resistance-related targets. STITCH was used to construct a network of compound–protein interactions, linking the phytochemicals to the relevant *Musca domestica* protein targets, including those involved in neurotransmission, metabolic regulation, and detoxification processes. A confidence score of 0.4 was applied to ensure the inclusion of high-confidence interactions. This step allowed for a comprehensive network pharmacology analysis to correlate bioactive compounds in the essential oils with key insecticide resistance proteins. Following this, GO enrichment analysis was performed using tools like STRING (https://string-db.org).

#### Molecular docking studies

To validate the network pharmacology predictions, molecular docking simulations were carried out to assess the binding interactions of key bioactive compounds with insect target proteins, such as acetylcholinesterase (AChE). Docking studies were performed using AutoDock Vina integrated with PyMOL for visual analysis, intending to identify promising compounds that exhibit high affinity for the selected targets^31^. The protein structures used for docking were derived from available PDB entries (AChE from *Drosophila melanogaster*, PDB ID: 6XYS), after preparing the proteins by removing water molecules, adding hydrogen atoms, and optimizing the structure using UCSF Chimera. The top docking poses were selected based on the strongest binding interactions, minimal RMSD (root-mean-square deviation), and favorable electrostatic/hydrophobic interactions. This docking process identified several promising compounds, including those that specifically targeted the AChE catalytic site.

####  Molecular dynamics simulation

To further evaluate the stability and binding affinity, molecular dynamics (MD) simulations were conducted using the GROMACS platform (version 2023)^32^. The protein-ligand complexes were prepared in UCSF Chimera, where hydrogen atoms were added to ensure proper protonation, and the complex was solvated in a TIP3P water box with a minimum distance of 1 nm between the solute and the box boundary to mimic physiological conditions. Sodium chloride (NaCl) ions were added to neutralize the system and maintain physiological salt concentration (150 mM). The CHARMM36 force field was applied for protein molecules, and CGenFF was used to parameterize the ligands. After initial energy minimization, the system underwent two equilibration phases: the NVT ensemble (constant volume and temperature) at 300 K using the V-rescale thermostat and the NPT ensemble (constant pressure and temperature) at 1.0 bar using the Berendsen barostat. Following equilibration, a 100-ns production MD simulation was performed to analyze the structural stability and ligand-receptor interactions. Trajectory analysis was conducted by calculating the root-mean-square deviation (RMSD) and radius of gyration (Rg) to assess the overall stability of the protein-ligand complex over time. Binding energy fluctuations were monitored to further assess the interaction strength.

### Statistical analysis

The obtained toxicity data were fitted to the log-probit model according to Hewlett 1972^33^ using an LDP line program (Ehab Soft, http://www.ehabsoft.com/ldpline), and LC_50_ and LC_90_ were determined for each material. The toxicity index was calculated as follows: LC_50_ (of the most effective material) / LC_50_ (of the required material) × 100.

## Results

### GC/MS profiling of *Citrus* species peel oils

#### *Citrus limon*

**Fig. 1:**
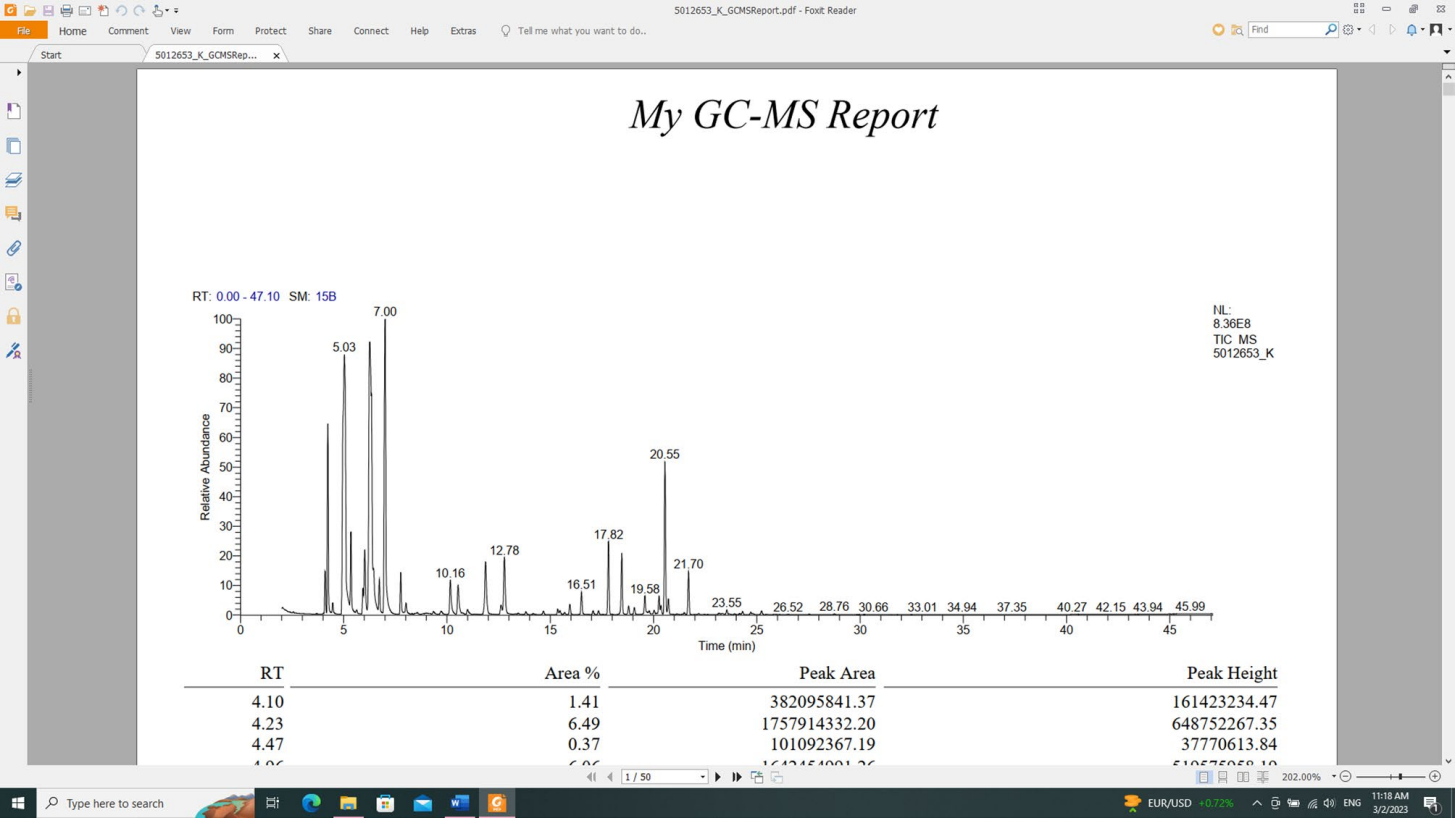
GC/MS spectra for *Citrus limon* peel oil.

C. limon peels yielded 2% v/w volatile oil based on fresh weight, which is colourless, possesses a distinctive aroma, is less dense than water, and is clear, transparent, and non-viscous at both room temperature and 4˚C. GC/MS analysis identified **21** chemicals, comprising 99.70% of all detected compounds, with 0.3% remaining unidentified (Table 1; Fig. 1).

**Fig. 2:**
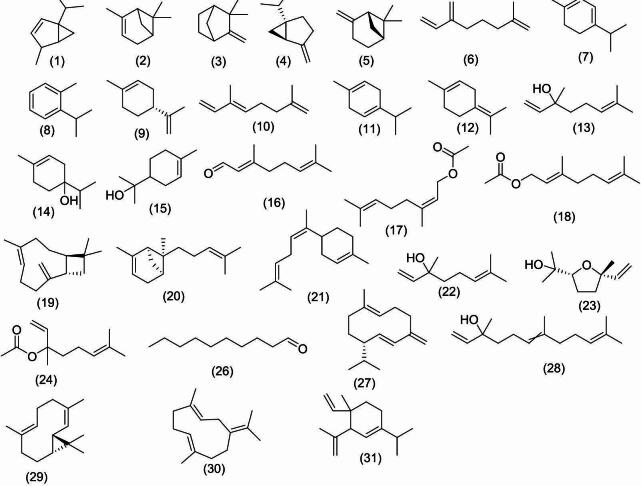
Compounds identified in the GC/MS analysis of the three *Citrus* species.

Using GC/MS analysis, the identified compounds belong to the monoterpene, aromatic hydrocarbon, and sesquiterpene classes. The monoterpenes class represented 85.99% of the total identified compounds, followed by sesquiterpenes with a percentage of 9.68%, and aromatic hydrocarbons with a percentage of 4.03%. Seventeen monoterpenes compounds (85.99%) were identified; ranging from cyclic hydrocarbon (36.11%) [*α*-terpinene **(7)**, D-limonene **(9)**, *γ*-terpinene **(11)**, *p*-mentha-1,4 (8)-diene **(12)**] which represented the major oil fraction, to oxygenated cyclic hydrocarbon (3.07%) [terpinen-4-ol **(14)**, *α*-terpineol **(15)**], and oxygenated acyclic hydrocarbon (10.13%) [3,7-dimethylocta-1,6-dien-3-ol **(13)**, *α*-citral **(16)**, nerol acetate **(17)**, geranyl acetate **(18**)], acyclic hydrocarbon (7.56%) [*α*-myrcene **(6)**, *α*-ocimene **(10)**] to bicyclic hydrocarbon (29.12%) [2-thujene **(1)**, α-pinene **(2)**, camphene **(3)**, (+)-sabinene **(4)**, (+)-*β*-pinene (**5**)]. While aromatic hydrocarbons represented (4.03%) of the total identified compounds, in the form of *o*-cymene (**8).** Also, three sesquiterpene compounds (9.68%) were identified, varying from cyclic hydrocarbon (4.86%), *cis*-α-bisabolene **(21)**, to a bicyclic hydrocarbon (4.82%) [caryophyllene (**19)**, *cis*-α-bergamotene (**20**)] (Table 1; Figs. 1, 2).

**Table 1:** *Citrus limon* peel oil composition using GC/MS analysis.

Identified compound	MF	%Area	RT	RI
2-Thujene (1)	C_10_H_16_	2.62	4.10	935
*α*-Pinene (2)	C_10_H_16_	7.49	4.23	924
Camphene (3)	C_10_H_16_	0.31	4.47	951
(+)-Sabinene (4)	C_10_H_16_	5.52	4.96	953
(+)-*β*-Pinene (5)	C_10_H_16_	13.18	5.03	929
*α*-Myrcene (6)	C_10_H_16_	7.05	5.35	981
*α-*Terpinene (7)	C_10_H_16_	0.41	5.93	939
*o-*Cymene (8)	C_10_H_14_	4.03	6.01	933
D-Limonene (9)	C_10_H_16_	15.46*	6.25	933
*α*-Ocimene (10)	C_10_H_16_	0.51	6.72	930
*γ-*Terpinene (11)	C_10_H_16_	17.51	7.00	931
*p-*Mentha-1,4(8)-diene (12)	C_10_H_16_	2.73	7.76	923
3,7-Dimethylocta-1,6-dien-3-ol (13)	C_10_H_18_O	0.80	8.01	925
Terpinen-4-ol (14)	C_10_H_18_O	1.34	10.16	933
α-Terpineol (15)	C_10_H_18_O	1.73	10.53	945
α-Citral (16)	C_10_H_16_O	5.99	11.86	908
Neryl acetate (17)	C_12_H_20_O_2_	1.82	15.95	924
Geranyl acetate (18)	C_12_H_20_O_2_	1.52	16.51	935
Caryophyllene (19)	C_15_H_24_	1.74	17.82	939
*cis*-α-Bergamotene (20)	C_15_H_24_	3.08	18.46	948
*cis*-*α*-Bisabolene (21)	C_15_H_24_	4.86	20.36	939
Monoterpenes hydrocarbon		85.99		
Aromatic hydrocarbon		4.03		
Sesquiterpene hydrocarbon		9.68		
Unidentified compounds		0.3		
Total		100.00%		

RI, retention index relative to *n*-alkanes; RT, retention time (min); MF, molecular formula; *major compound; %: percentage.

#### *Citrus aurantium*

**Fig. 3:**
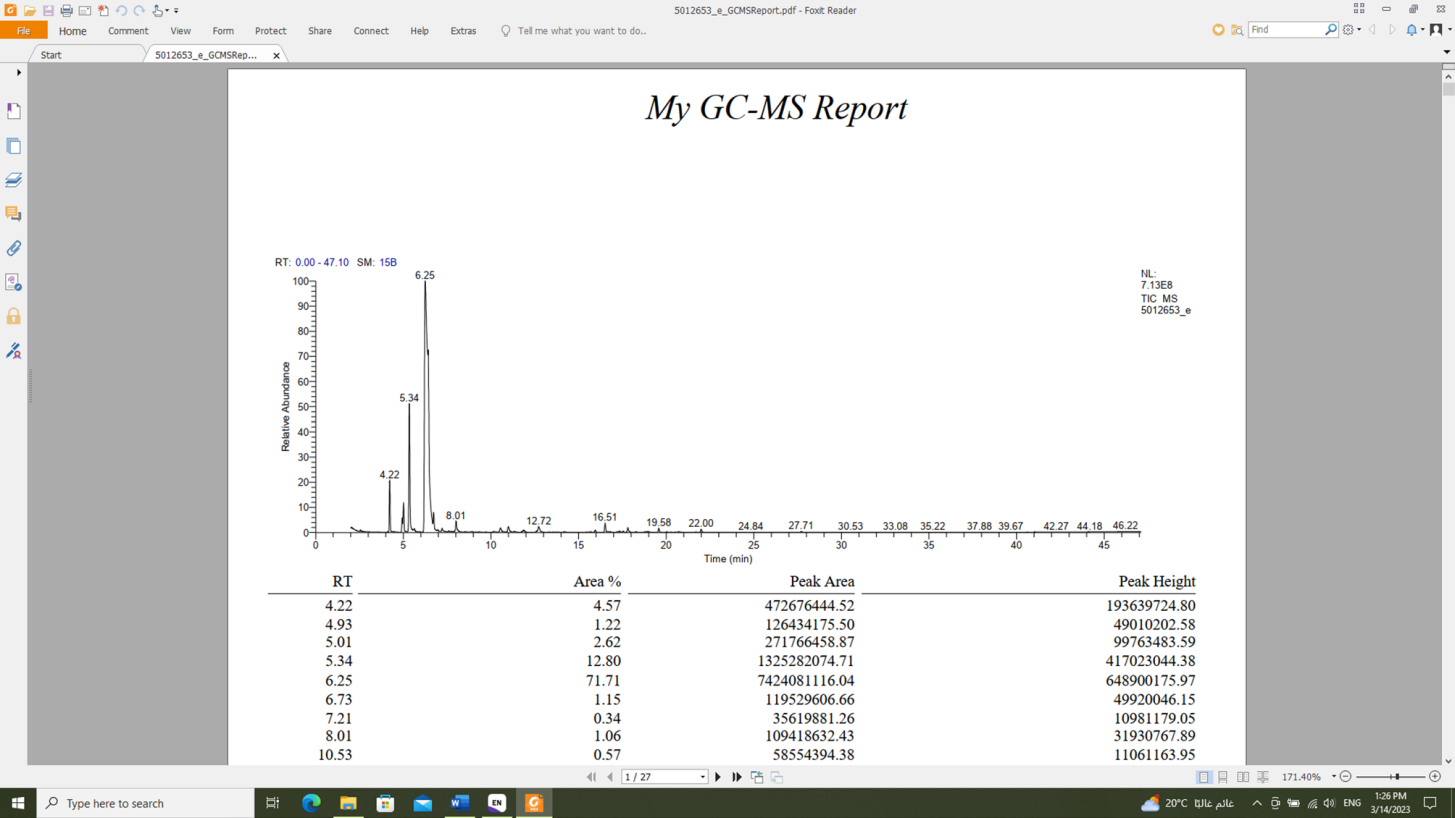
GC/MS spectra for *Citrus aurantium* peel oil.

*C. aurantium* peels gave 4% v/w volatile oil fresh weight, being colourless with a characteristic odor, lighter than water, clear, transparent, and not viscous at room temperature as well as at 4 °C. GC/MS analysis identified a total of **15** compounds, which accounted for 100% of all compounds detected. The identified compounds belonged to various chemical classes, including monoterpenes and sesquiterpenes. The monoterpenes represented 98.71% of the total identified compounds, followed by sesquiterpenes (1.29%). Twelve monoterpenes compounds (98.71%) were identified; ranging from cyclic hydrocarbon (71.71%) [D-limonene **(9)**] which represented the major oil fraction, oxygenated cyclic hydrocarbon ( 0.34%) [*cis*-linalool oxide (**23)**], to oxygenated acyclic hydrocarbon (3.73%) [linalool **(22)**, linalyl acetate **(24)**, geranyl acetate **(18)**, decanal **(26)**], acyclic hydrocarbon (13.95%) [*α*-myrcene **(6)**, α-ocimene **(10)**], to bicyclic hydrocarbon (8.41%) [α-pinene (**2**), (+)-sabinene **(4)**, (+)-*β*-pinene **(5)**], and oxygenated bicyclic hydrocarbon (0.57%) [(-)-(1 S,2R,4R)-*β*-fenchol] **(25)**. Also, three sesquiterpene compounds (1.29%) were identified, varying from a mono and bicyclic hydrocarbon [caryophyllene **(19)**, germacrene D **(27)**], to an oxygenated cyclic hydrocarbon [nerolidol **(28)**] (Table 2; Figs. 2, 3).

**Table 2:** *Citrus aurantium* peel oil composition using GC/MS analysis.

Identified compound	MF	%Area	RT	RI
*α*-Pinene (2)	C_10_H_16_	4.57	4.22	924
(+)-Sabinene (4)	C_10_H_16_	1.22	4.96	953
(+)-*β*-Pinene (5)	C_10_H_16_	2.62	5.03	929
*α*-Myrcene (6)	C_10_H_16_	12.80	5.34	981
D-Limonene (9)	C_10_H_16_	71.71*	6.25	933
α-Ocimene (10)	C_10_H_16_	1.15	6.73	918
Linalool (22)	C_10_H_18_O	1.06	8.01	923
*cis*-Linalool oxide (23)	C_10_H_18_O_2_	0.34	7.21	900
Linalyl acetate (24)	C_12_H30O_2_	0.87	12.72	906
Geranyl acetate (18)	C_12_H_20_O_2_	1.05	16.51	931
(-)-(1 S,2R,4R)-*β*-Fenchol (25)	C_10_H_18_O	0.57	10.52	940
Decanal (26)	C_10_H_20_O	0.75	10.99	936
Caryophyllene (19)	C_15_H_24_	0.54	17.82	939
Germacrene D (27)	C_15_H_24_	0.43	19.58	958
Nerolidol (28)	C_15_H_26_O	0.32	22.00	926
Monoterpenes hydrocarbon		94.07		
Oxygenated monoterpenes hydrocarbon		4.64		
Sesquiterpene hydrocarbon		0.97		
Oxygenated sesquiterpene hydrocarbon		0.32		
Total		100.00%		

RI, retention index relative to *n*-alkanes; RT, retention time (min); MF, molecular formula; *major compound; %: percentage.

#### *Citrus margarita*

**Fig. 4:**
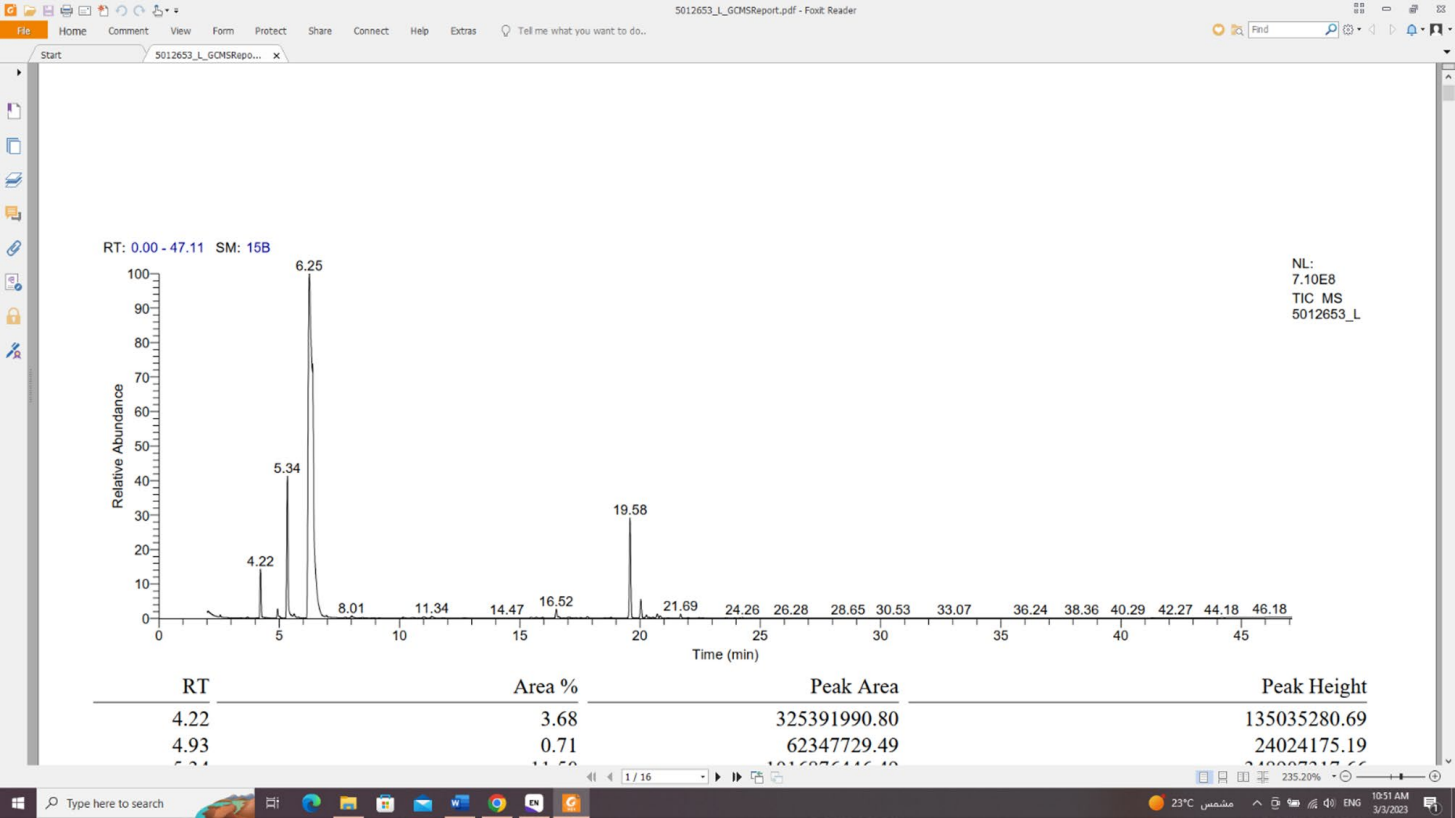
GC/MS spectra for *Citrus margarita* peel oil.

*Citrus margarita* peels gave 6.0% v/w volatile oil fresh weight, being colourless with a characteristic odor, lighter than water, clear, transparent, and not viscous at room temperature as well as at 4 °C. GC/MS analysis was used to identify a total of **9** compounds, accounting for 100.00% of all compounds found. The identified compounds belonged to different chemical classes, including monoterpene and sesquiterpene. The monoterpenes class represented (88.31%) of the total identified compounds, followed by sesquiterpenes (11.69%). Five monoterpenes’ compounds were identified; ranging from cyclic hydrocarbon (71.59%) [D-limonene **(9)**], which represented the major oil fraction, to oxygenated acyclic hydrocarbon (0.83%) [neryl acetate **(17)**], acyclic hydrocarbon (11.50%) [*α*-myrcene **(6)**], to bicyclic hydrocarbon (4.39%) [*α*-pinene **(2)** and (+)-sabinene **(4)**]. Also, four sesquiterpene compounds (11.69%) were identified, varying from cyclic hydrocarbon (10.00%) [germacrene D **(27)**, germacrene B **(30)**, 3-isopropenyl-1-isopropyl-4-methyl-4-vinyl-1-cyclohexene **(31)**], to bicyclic hydrocarbon (1.69%) [lepidozene **(29)**] (Table 3; Figs. 2, 4).

**Table 3:** *Citrus margarita* peel oil composition using GC/MS analysis.

Identified compound	MF	%Area	RT	RI
*α*-Pinene (2)	C_10_H_16_	3.68	4.22	924
(+)-Sabinene (4)	C_10_H_16_	0.71	4.96	953
*α*-Myrcene (6)	C_10_H_16_	11.50	5.34	981
D-Limonene (9)	C_10_H_16_	71.59*	6.25	933
Neryl acetate (17)	C_12_H_20_O_2_	0.83	15.95	924
Germacrene D (27)	C_15_H_24_	9.28	19.58	958
Lepidozene (29)	C_15_H_24_	1.69	20.03	908
Germacrene B (30)	C_15_H_24_	0.35	21.69	947
3-Isopropenyl-1-isopropyl-4-methyl-4-vinyl-1-cyclohexene (31)	C_15_H_24_	0.37	20.72	906
Monoterpenes hydrocarbon		87.48		
Oxygenated monoterpenes hydrocarbon		0.83		
Sesquiterpene hydrocarbon		11.69		
Total		100.00%		

RI, retention index relative to *n*-alkanes; RT, retention time (min); MF, molecular formula; *major compound; %: percentage.

### The toxicity of *Citrus limon*, *Citrus aurantium*, and *Citrus margarita* peel oils against *Musca domestica* adults

**Fig. 5:**
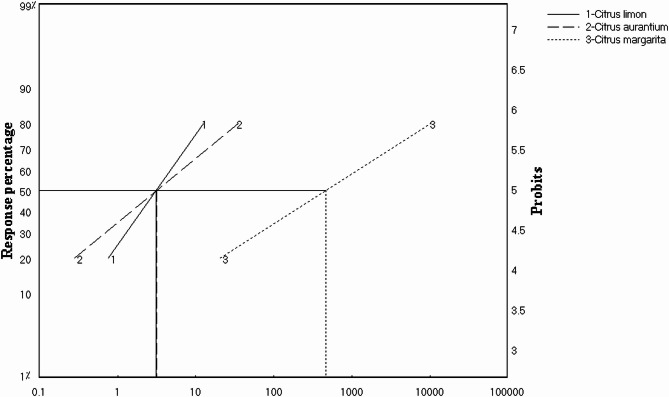
Toxicity lines of *Citrus limon*, *Citrus aurantium*, and *Citrus margarita* peel oils against *Musca domestica* adults showing LC_50_ differences. The obtained toxicity data were fitted to the log-probit model according to Hewlett 1972^33^ using an LDP line program (Ehab Soft, http://www.ehabsoft.com/ldpline), and LC_50_ and LC_90_ were determined for each material. The toxicity index was calculated as follows: LC_50_ (of the most effective material) / LC_50_ (of the required material) × 100. All experiments were performed at least three times.

As shown in Table 4, *C. limon* oil was more effective against housefly adults than *C. aurantium* and *C. margarita* oils, with LC_50_ values of 3.19, 3.25, and 479.12 ppm, respectively. Therefore, the results showed that *C. limon* was the most effective oil tested against housefly adults, with a 100% toxicity index and an LC_50_ of 3.19 ppm, while *C. C. aurantium* was nearly as effective as *C. limon* oil, with an LC_50_ of 3.25 ppm with a 98.093% toxicity index, but *C. limon* was still stronger, as its slope was higher than *C. aurantium*, showing 1.381 and 0.802 for *C. limon* and *C. aurantium*, respectively. While *C. margarita* was the weakest tested oil, as its toxicity index was 0.666% in comparison to *C. limon* and *C. aurantium* (Tables S3–S5, Fig. 5).

**Table 4:** The efficacy data of *Citrus limon*,* Citrus aurantium*, and *Citrus margarita* peel oils against *Musca domestica* adults.

Scientific name	LC_50_ (ppm)	LC_90_ (ppm)	Toxicity index (%)	Slope
*Citrus limon*	3.19	27.05	100	1.38
*Citrus aurantium*	3.25	129.0	98.09	0.802
*Citrus margarita*	479.12	5486.5	0.666	0.6225

The obtained toxicity data were fitted to the log-probit model according to Hewlett 1972^33^ using an LDP line program (Ehab Soft, http://www.ehabsoft.com/ldpline), and LC_50_ and LC_90_ were determined for each material. The toxicity index was calculated as follows: LC_50_ (of the most effective material)/LC_50_ (of the required material) × 100. All experiments were performed at least three times.

### Therapeutic targets for *Musca domestica* insecticide resistance

**Fig. 6:**
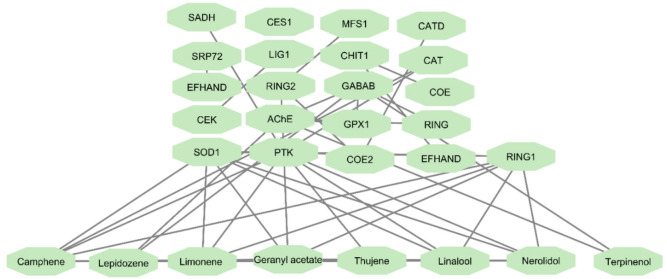
STITCH interactions for *Musca domestica* insecticide resistance. The STITCH-derived compound–protein interaction network was imported into Cytoscape for visualization, showing interactions between bioactive compounds from citrus peel oils and insecticide resistance–related proteins in *Musca domestica*, including AChE, GABA receptors, and cytochrome P450 enzymes. The network highlights notable interactions involving D-limonene, geranyl acetate, and α-terpineol (terpineol) with these resistance-associated targets, supporting their potential roles in modulating neurotransmission and detoxification pathways.

The STITCH analysis (Fig. 6) revealed significant interactions between key compounds, such as D-limonene, geranyl acetate, and α-terpineol (terpineol), with *Musca domestica* protein targets, including AChE, GABA receptors, and cytochrome P450 enzymes. Consistent with Fig. 6, the network highlights notable interactions involving D-limonene, geranyl acetate, and α-terpineol (terpineol) with these resistance-associated targets. These interactions suggest that these bioactive compounds may modulate insecticide resistance pathways in *Musca domestica*, influencing key biological processes such as neurotransmission and detoxification. Interactions with AChE and GABA receptors support a plausible contribution to neurotransmission-related mechanisms, whereas links to cytochrome P450 enzymes indicate potential effects on metabolic detoxification pathways. Collectively, the connectivity pattern of D-limonene, geranyl acetate, and α-terpineol suggests that these bioactives may influence resistance through complementary modes of action, warranting emphasis as key candidates for downstream mechanistic interpretation and experimental validation.

**Fig. 7:**
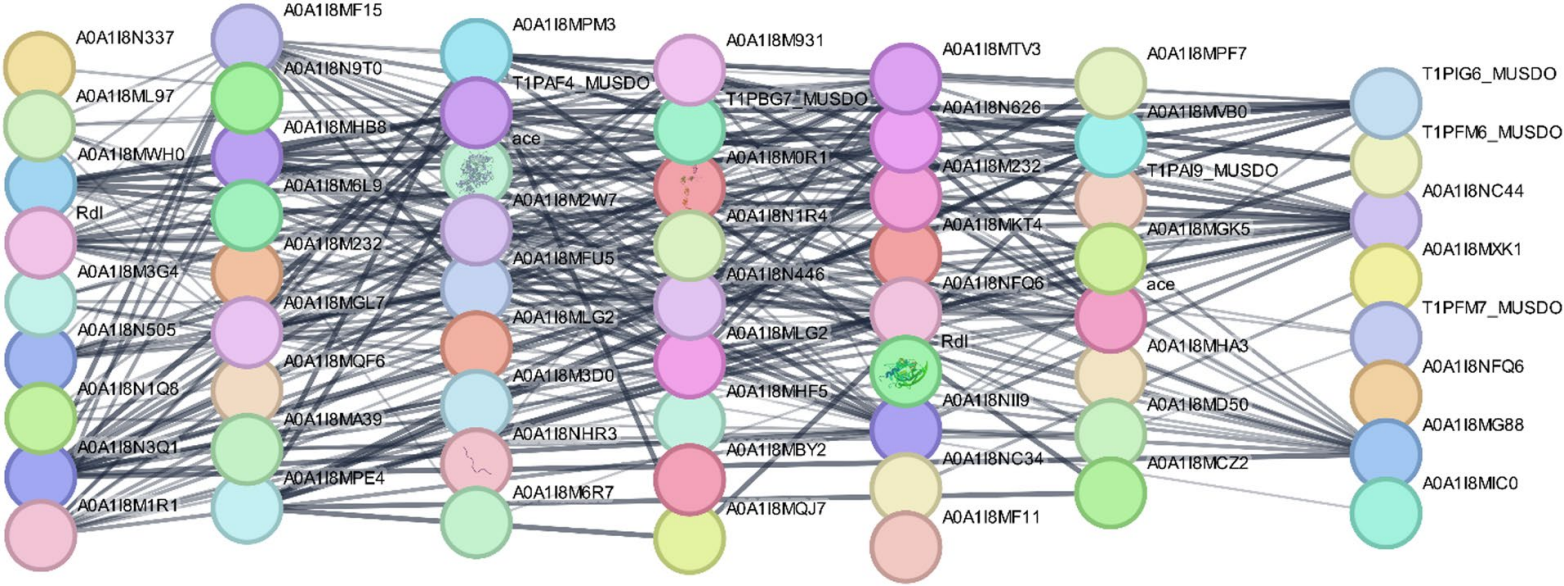
STRING network analysis of protein–protein interactions for *Musca domestica* insecticide resistance. The network includes multiple protein targets involved in neurotransmission, detoxification, and metabolic regulation, which interact with bioactive compounds from citrus species.

To construct a PPI network that elucidates the interactions between insecticide resistance-related proteins and bioactive compounds identified from citrus essential oils, we utilized the STRING database (version 12.0). This analysis aimed to uncover key molecular targets influenced by the bioactive compounds and their potential role in modulating resistance pathways in *Musca domestica*. The network was visualized using Cytoscape (version 3.10.3), which integrated both PPIs and compound–protein interactions. As shown in Fig. 7, the network consists of 61 nodes representing key proteins involved in neurotransmission, detoxification, and metabolic regulation, as well as compounds identified from citrus essential oils. A total of 277 interaction linkages were observed, with an average node connectivity of 11.42, suggesting strong functional correlations between the insecticide resistance targets and bioactive compounds. Table 4 summarizes the key protein targets identified in the network, their functional categories, and the specific bioactive compounds that interact with these proteins in the context of insecticide resistance.

**Table 4:** Key protein targets and functional categories in *Musca domestica* resistance pathways.

Node_ID	Protein name	Functional bucket
Ace	Carboxylic ester hydrolase; Acetylcholinesterase-like; type-B carboxylesterase/lipase family	Neurotransmission/cholinergic
A0A1I8MHF5	Uncharacterized protein; sodium: solute symporter (SSF, TC 2.A.21) family	Transporter/solute carrier
A0A1I8M3G4	MFS domain-containing protein	Transporter/MFS family
A0A1I8MLG2	Uncharacterized protein; ligand-gated ion channel (TC 1.A.9) family	Neurotransmission/ion channel
Rdl	GABA receptor subunit beta; ligand-gated chloride channel (TC 1.A.9.5)	Neurotransmission/GABA
A0A1I8MIC0	MFS domain-containing protein	Transporter/MFS family
A0A1I8NFQ6	Uncharacterized protein; ligand-gated ion channel (TC 1.A.9) family	Neurotransmission/ion channel
A0A1I8MF15	Catalase	Detox/redox
A0A1I8NII9	Catalase domain-containing protein	Detox/redox
A0A1I8MD50	Glutathione peroxidase	Detox/redox
A0A1I8MA39	Superoxide dismutase [Cu-Zn]	Detox/redox
A0A1I8M232	Uncharacterized protein; Cytochrome P450 family	Detox/xenobiotic metabolism
T1PFM7_MUSDO	S-(hydroxymethyl)glutathione dehydrogenase; Class-III ADH	Detox/alcohol metabolism
T1PBG7_MUSDO	Biopterin-dependent aromatic amino acid hydroxylase	Neurotransmitter biosynthesis
A0A1I8N446	Carn_acyltransferase domain-containing protein	Metabolism/acetyl-transfer
A0A1I8MTV3	Carn_acyltransferase domain-containing protein	Metabolism/acetyl-transfer
A0A1I8MPM3	Uncharacterized protein	Uncharacterized/Unknown
A0A1I8MPE4	Uncharacterized protein	Uncharacterized/Unknown
A0A1IMVB0	Uncharacterized protein	Uncharacterized/Unknown
A0A1IMHA3	Signal recognition particle subunit SRP72	Protein trafficking/SRP pathway
T1PAI9_MUSDO	Chitin-binding protein	Cuticle/structural
A0A1IMBY2	Uncharacterized protein; protein kinase superfamily (Tyr kinase family)	Signaling/kinase
A0A1IMQF6	Uncharacterized protein	Uncharacterized/unknown
A0A1IN9T0	EF-hand domain-containing protein	Calcium-binding/signaling
A0A1IMXK1	RING-type domain-containing protein	Protein degradation/ubiquitin ligase
A0A1INC34	RING-type domain-containing protein	Protein degradation/ubiquitin ligase
A0A1IMKT4	DNA ligase	DNA repair/replication
A0A1IMF11	Aminopeptidase	Proteolysis
A0A1IM0R1	Uncharacterized protein	Uncharacterized/unknown
A0A1INHR3	A4_EXTRA domain-containing protein	Immune/extracellular matrix
A0A1IMGL7	Carn_acyltransferase domain-containing protein	Metabolism/acetyl-transfer
A0A1IMCZ2	COesterase domain-containing protein	Detox/ester metabolism
A0A1IMQJ7	Carboxylic ester hydrolase; type-B carboxylesterase/lipase family	Detox/ester metabolism
A0A1IM3D0	Carboxylic ester hydrolase; type-B carboxylesterase/lipase family	Detox/ester metabolism
A0A1IMGK5	COesterase domain-containing protein	Detox/ester metabolism
A0A1IMPF7	Carboxylic ester hydrolase; type-B carboxylesterase/lipase family	Detox/ester metabolism
T1PFM6_MUSDO	Choline/ethanolamine kinase	Lipid metabolism/signaling

Table 4 presents the key protein targets involved in insecticide resistance pathways in *Musca domestica*, showing their functional roles and the interactions with bioactive compounds from citrus essential oils. The compounds, including D-limonene, geranyl acetate, and terpinenol, demonstrated significant interactions with several proteins critical to neurotransmission, detoxification, and metabolic regulation. These interactions suggest that the identified compounds might play an essential role in modulating resistance pathways, influencing key biological processes involved in neurotransmission, detoxification, and immune modulation.

**Fig. 8:**
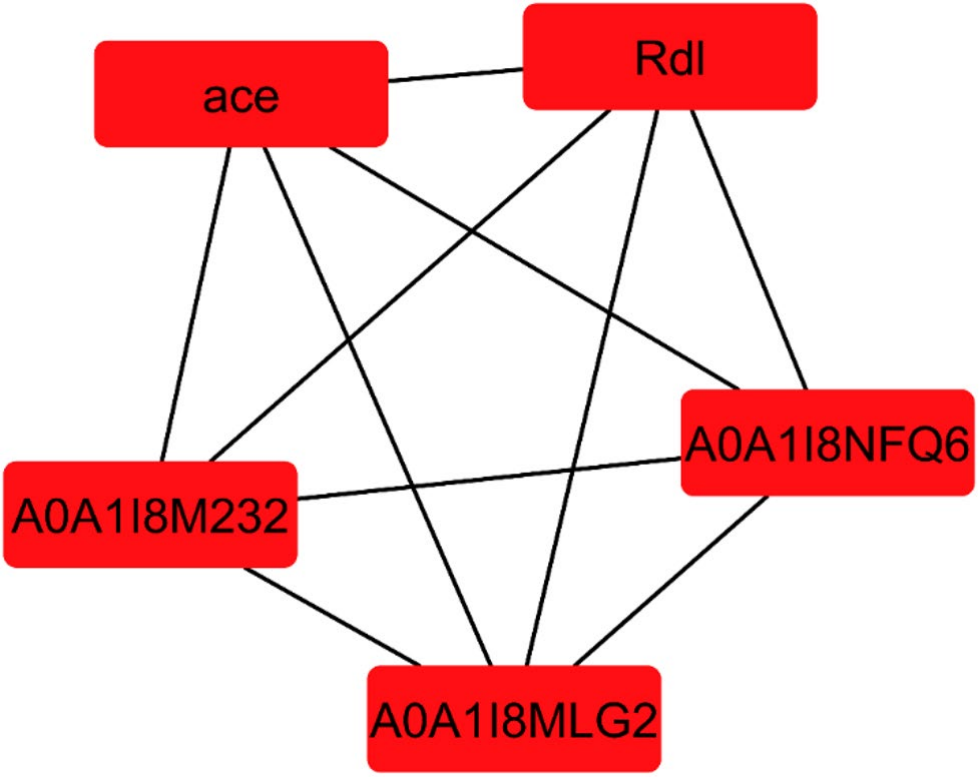
Hub genes in the protein–protein interaction network for *Musca domestica* insecticide resistance. PPI network analysis was conducted using the CytoHubba plugin to identify key hub genes involved in insecticide resistance pathways in *Musca domestica*. The network highlights critical insecticide resistance-related proteins such as acetylcholinesterase (AChE), GABA receptors, cytochrome P450 enzymes, and glutathione S-transferase (GST), all of which interact with bioactive compounds from citrus species.

Using the CytoHubba plugin in Cytoscape, key hub genes were identified based on their high connectivity within the network. The hub genes in the network are critically involved in neurotransmission, detoxification, and metabolic regulation—pathways that are pivotal in insecticide resistance mechanisms (Fig. 8). Proteins like acetylcholinesterase (AChE), GABA receptors, cytochrome P450 enzymes, and glutathione S-transferase (GST) emerged as significant targets due to their roles in neurotransmission and detoxification. These proteins are well-known for their involvement in pesticide resistance, especially by altering the metabolism and detoxification of insecticides. The bioactive compounds from citrus oils showed strong interactions with these proteins, suggesting their potential role in modulating insecticide resistance pathways.

### Analysis of enriched gene ontology (GO) terms

**Fig. 9:**
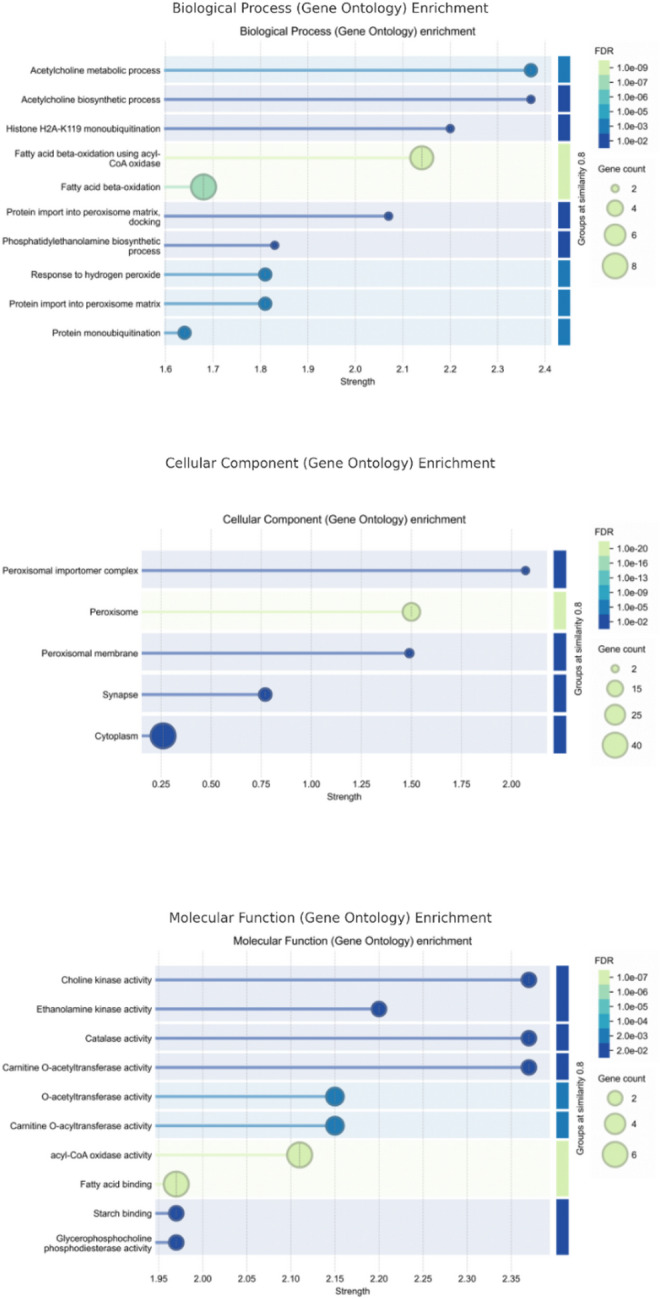
Bubble plot of GO enrichment analysis of bioactive compounds from *Citrus* species in insecticide resistance mechanisms. GO enrichment analysis of biological processes (BP), cellular components (CC), and molecular functions (MF) associated with insecticide resistance in *Musca domestica* (STRING-based enrichment). Bubble size indicates the number of genes involved, color denotes the false discovery rate (FDR), and the x-axis reflects enrichment signal strength, while GO terms are shown on the y-axis.

To investigate the molecular mechanisms underlying the insecticidal potential of bioactive compounds from *Citrus* species (*C. limon*, *C. aurantium*, and *C. margarita*) against insecticide resistance in *Musca domestica*. A Gene Ontology (GO) enrichment analysis was performed using STRING. This analysis identified key biological processes (BP), cellular components (CC), and molecular functions (MF) associated with insecticide resistance mechanisms and the immune response in the context of pesticide exposure (Fig. 9). The biological process (BP) enrichment analysis highlighted significant pathways, such as the acetylcholine metabolic process and fatty acid beta-oxidation. These pathways are essential for neurotransmission, energy production, and immune response, all of which are critical for the detoxification of insecticides and resistance to insecticide-induced stress. The involvement of acetylcholine metabolism suggests that the bioactive compounds from citrus species may affect neurotransmission, which is directly targeted by many neurotoxic insecticides. Similarly, fatty acid beta-oxidation is important for energy production, which is necessary for detoxification processes and resistance mechanisms. These findings suggest that citrus bioactive compounds may modulate both neurological and metabolic pathways crucial for overcoming insecticide resistance. In the cellular component (CC) category, the analysis identified significant terms such as peroxisomal membrane and synaptic vesicle. These components are involved in detoxification, protein synthesis, and neurotransmission, all of which play a vital role in insecticide resistance. Peroxisomal membranes are particularly important as they are involved in oxidative stress management by detoxifying reactive oxygen species (ROS) produced during insecticide exposure. Synaptic vesicles, essential for neurotransmission, are directly targeted by neurotoxic insecticides, indicating that the bioactive compounds from citrus species may modulate neurosignaling, potentially reducing the toxicity caused by neurotoxic insecticides. The molecular function (MF) enrichment analysis identified significant terms such as choline kinase activity and catalase activity. These functions are vital for membrane signaling, detoxification, and oxidative stress management. Choline kinase activity is involved in the synthesis of acetylcholine, a neurotransmitter targeted by many insecticides, suggesting that the bioactive compounds from citrus species may help modulate neurotransmission and improve resistance to neurotoxic insecticides.

### Analysis of enriched KEGG pathways

**Fig. 10:**
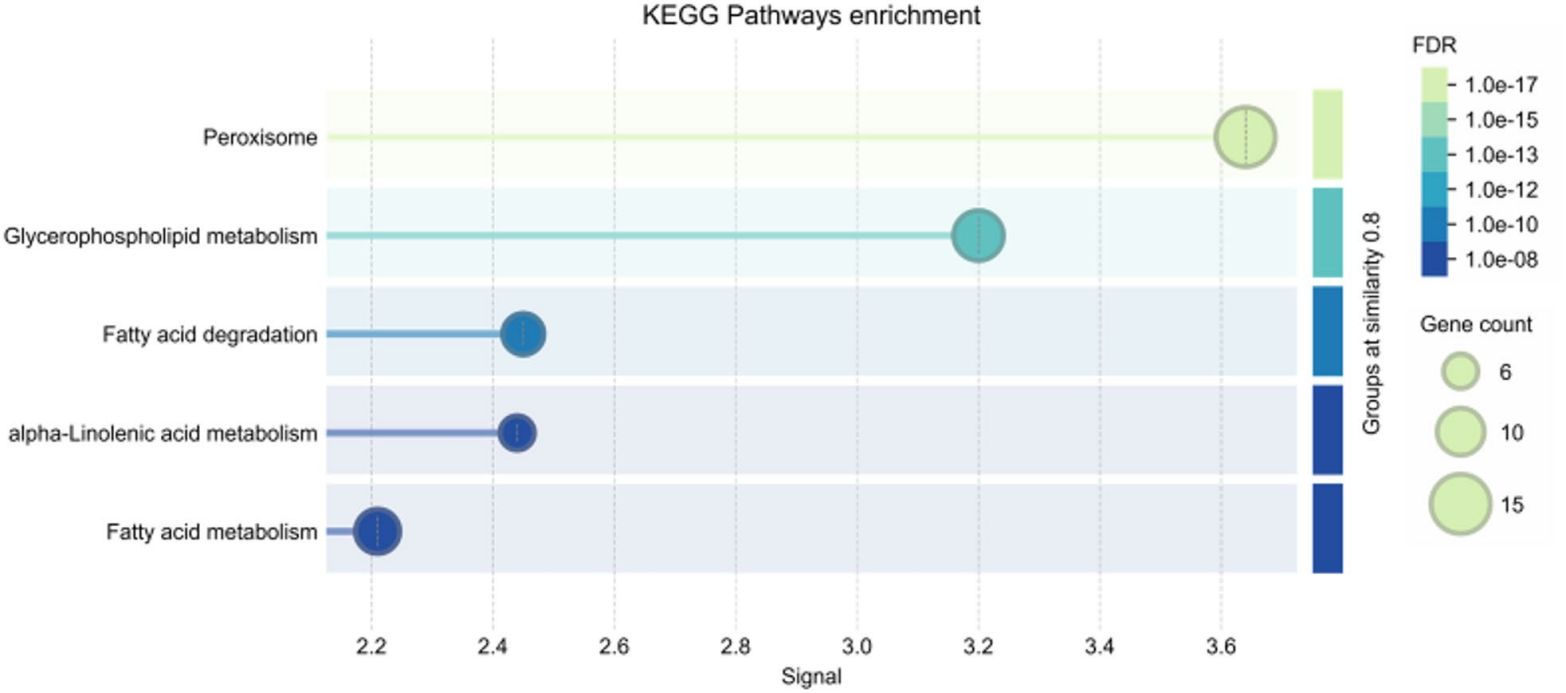
Bubble plot of KEGG pathway enrichment of bioactive compounds from *Citrus* species in insecticide resistance. Pathway enrichment analysis was performed using the STRING database. Bubble size indicates the number of genes involved, color denotes the false discovery rate (FDR), and the x-axis reflects enrichment signal strength.

To understand the molecular mechanisms underlying the insecticidal potential of bioactive compounds from *Citrus* species (*C. limon*, *C. aurantium*, and *C. margarita*) against insecticide resistance in *Musca domestica*, a KEGG pathway enrichment analysis was conducted^34,35^. This analysis aimed to identify key metabolic pathways involved in detoxification, oxidative stress management, and energy production, which play a crucial role in how these compounds could kill insects by targeting specific biological processes associated with insecticide resistance. The KEGG enrichment analysis highlighted several critical pathways that are essential for insect survival and resilience in the face of insecticide exposure^36^. The most significant pathways identified were fatty acid metabolism, glycerophospholipid metabolism, and peroxisomal function, all of which are involved in detoxification, energy production, and oxidative stress management (Fig. 10). In the fatty acid metabolism pathway, several key processes, such as fatty acid degradation, alpha-linolenic acid metabolism, and general fatty acid metabolism, were enriched. These processes are essential for energy production and detoxification. The breakdown of fatty acids produces energy that is crucial for maintaining insect survival, especially when exposed to neurotoxic insecticides that may disrupt normal metabolic functions. The disruption of fatty acid metabolism can result in energy depletion, making the insect more susceptible to insecticide-induced toxicity. Additionally, alpha-linolenic acid metabolism is involved in managing oxidative stress, which is elevated during insecticide exposure. By interfering with these metabolic pathways, the bioactive compounds from Citrus species may help overwhelm the insect’s ability to produce energy and detoxify, leading to insect mortality. The glycerophospholipid metabolism pathway, which is responsible for membrane integrity and neurotransmission, was also significantly enriched. This pathway is crucial for maintaining the structure and function of cell membranes, especially in neuronal cells. Neurotoxic insecticides, such as organophosphates and pyrethroids, target these pathways, leading to neurological damage in insects. By modulating glycerophospholipid metabolism, the bioactive compounds from Citrus species may disrupt neurotransmission and membrane integrity, enhancing the neurotoxic effects of insecticides. This disruption can result in paralysis and death of the insect. Overall, the KEGG pathway enrichment analysis suggests that the bioactive compounds from Citrus species may kill insects by targeting multiple pathways involved in energy production, detoxification, and neurotransmission.

### Molecular docking analysis

Acetylcholinesterase (AChE) was identified as a predominant target in the PPI network of our study, highlighting its crucial role in neurotransmission and insecticidal action. As a key enzyme responsible for the breakdown of acetylcholine, inhibition of AChE leads to the accumulation of this neurotransmitter, resulting in nerve dysfunction, paralysis, and ultimately, death in insects. Given its significance in insecticide resistance and its centrality in the network, AChE was chosen as a primary target for molecular docking studies to evaluate the binding affinity of the Citrus bioactive compounds and assess their potential as insecticidal agents. To explore the potential inhibitory interaction of bioactive compounds from Citrus species with AChE, molecular docking was conducted against AChE from *Drosophila melanogaster* (PDB ID: 6XYS), which shares homology with AChE2 in *Musca domestica*^37^. This approach aimed to identify promising natural insecticidal agents capable of interfering with the cholinergic system in insects. To ensure the reliability of the docking protocol, re-docking of the co-crystallized ligand (coligand) was performed into the active site of *Drosophila melanogaster* AChE (PDB ID: 6XYS). The resulting docking pose was compared to its original crystallographic conformation, yielding a root mean square deviation (RMSD) of 1.01 Å, which is within the acceptable threshold (< 2.0 Å) for docking validation. This low RMSD indicates that the docking parameters were suitably optimized and capable of accurately reproducing the native binding orientation of the ligand. Therefore, the docking protocol was considered reliable for further virtual screening of the citrus-derived compounds, ensuring that the predicted interactions and binding affinities reflect biologically relevant conformations. A total of **31** citrus-derived compounds were docked, and their binding affinities (S scores) and root mean square deviations (RMSD) were evaluated (Table S2). The molecular docking analysis of **31** citrus-derived compounds against *Drosophila melanogaster* acetylcholinesterase (AChE; PDB ID: 6XYS), which shares significant homology with insect AChE2, revealed diverse binding affinities ranging from − 6.06 to − 10.2 kcal/mol, with the following results: 2-thujene (– 7.12), α-pinene (– 8.85), camphene (– 8.2), (+)-sabinene (– 7.8), (+)-β-pinene (– 6.47), α-myrcene (– 6.47), α-terpinene (– 6.17), o-cymene (– 8.6), d-limonene (– 9.5), α-ocimene (– 8.12), *Υ*-terpinene (– 6.06), p-mentha-1,4(8)-diene (– 8.91), 3,7-dimethylocta-1,6-dien-3-ol (– 8.5), terpinen-4-ol (– 9.2), α-terpineol (– 6.55),α-citral (geranial) (– 6.55), neryl acetate (– 6.91), geranyl acetate (– 10.2), caryophyllene (– 7.3), cis-α-bergamotene (– 6.87), cis-α-bisabolene (– 7.84), linalool (– 6.42), cis-linalool oxide (– 6.88), linalyl acetate (– 7.1), (−)-(1s,2r,4r)-β-fenchol (– 7.37), decanal (– 8.36), germacrene D (– 6.6), nerolidol (– 7.54), lepidozene (– 7.78), germacrene B (– 6.14), and 3-isopropenyl-1-isopropyl-4-methyl-4-vinyl-1-cyclohexene (– 7.82). The control coligand showed a binding affinity of − 9.22 kcal/mol. rmsd values ranged between 0.56 and 2.25 A˚, indicating varied conformational stability across compounds.

**Fig. 11:**
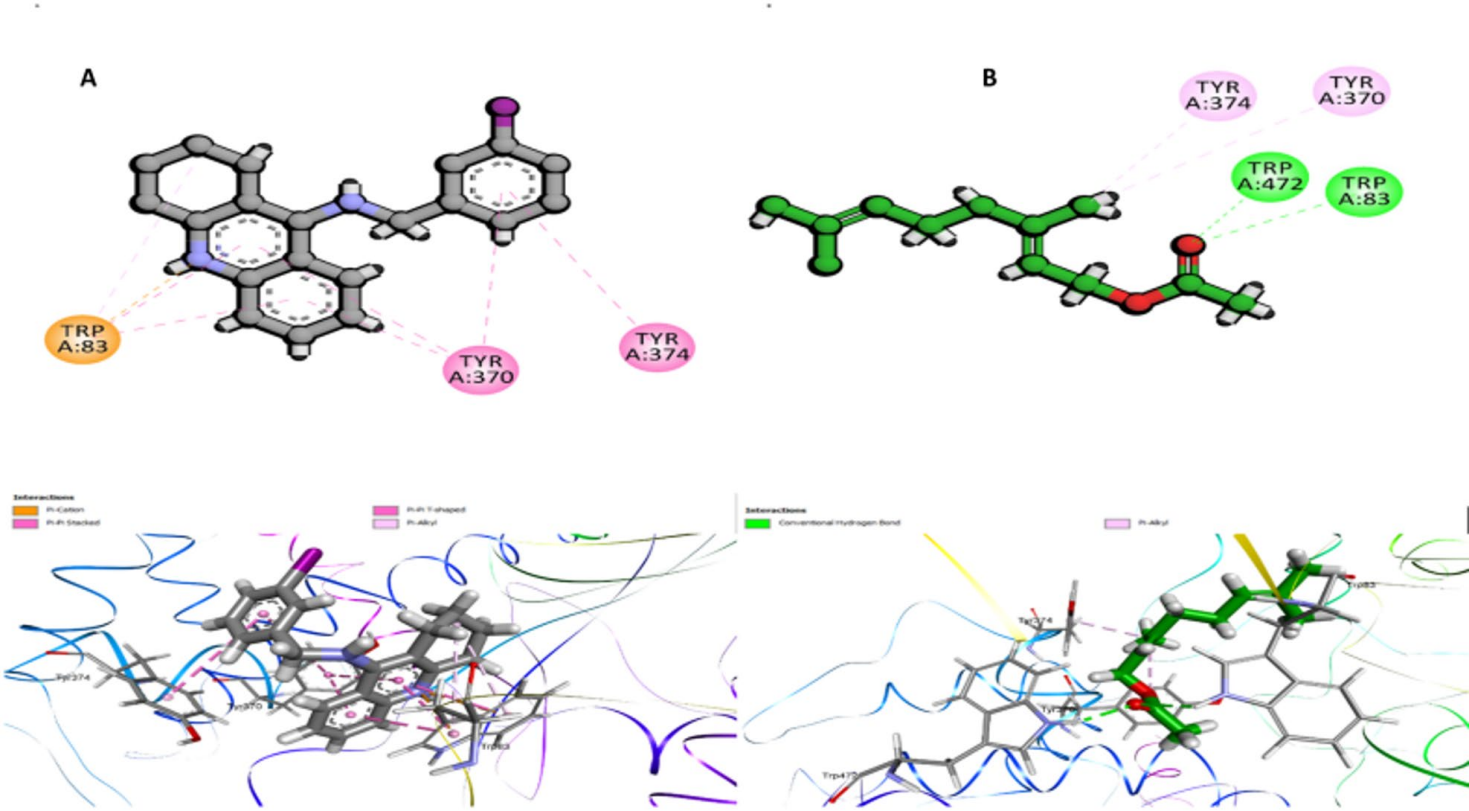
Docking interactions with AChE (PDB: 6XYS). (**A**) Co-crystallized ligand showing key interactions with TYR370, TYR374, and TRP83. (**B**) Compound **18** (geranyl acetate) forms similar interactions, including a hydrogen bond with TRP472, indicating strong and competitive binding with potential insecticidal relevance.

To ensure robust interpretation, compounds were filtered using two criteria: (1) docking scores below − 8.0 kcal/mol, indicating high binding affinity, and (2) RMSD values below 2.0 Å, suggesting stable and reproducible binding orientations. Based on these filters, several compounds—such as geranyl acetate (–10.2 kcal/mol, RMSD 0.62 Å), D-limonene (–9.5, 0.78 Å), terpinen-4-ol (–9.2, 0.89 Å), p-mentha-1,4(8)-diene (–8.91, 0.56 Å), and α-pinene (–8.85, 0.62 Å)—stood out as strong candidates. Among these, compound **18** (geranyl acetate) was prioritized for further analysis due to exhibiting the lowest binding energy and excellent pose stability, suggesting a strong and specific interaction with the AChE active site. Notably, this compound outperformed the reference coligand (co-crystallized inhibitor), which had an S score of − 9.22 kcal/mol and RMSD of 1.01 Å. The 2D and 3D binding poses revealed that the coligand forms π-π stacked and π-cation interactions with key residues Trp83, Tyr370, and Tyr374, which are critical in the ligand recognition pocket of AChE (Fig. 11A). These interactions are known to stabilize inhibitors at the catalytic gorge and hinder acetylcholine hydrolysis, ultimately leading to impaired neural function and insect paralysis. Notably, compound **18** (geranyl acetate) demonstrated a similar interaction profile, engaging with the same residues—TYR370, TYR374, and TRP83—and additionally forming a hydrogen bond with TRP472, as shown in both 2D and 3D visualizations (Fig. 11B). This overlap suggests that geranyl acetate targets the same active-site region as the coligand and may competitively inhibit AChE activity. The shared binding architecture between compound **18** and the coligand, particularly involving the conserved aromatic residues, reinforces the idea that compound **18** can act as a potent AChE inhibitor, thus contributing to the insecticidal mechanism of Citrus-derived compounds. This docking analysis not only highlights compound **18** as a promising lead molecule but also validates the computational pipeline. The consistency in interaction patterns further supports the role of geranyl acetate in modulating cholinergic transmission, offering a rational basis for its future development as a botanical insecticide.

### Molecular dynamics simulation analysis

To further assess the stability and dynamic behavior of the AChE–geranyl acetate complex, a 100 ns molecular dynamics (MD) simulation was performed. Several key metrics, including root-mean-square deviation (RMSD), root-mean-square fluctuation (RMSF), radius of gyration (Rg), hydrogen bonding, and potential energy, were evaluated to elucidate the structural stability and binding persistence of the complex.

**Fig. 12:**
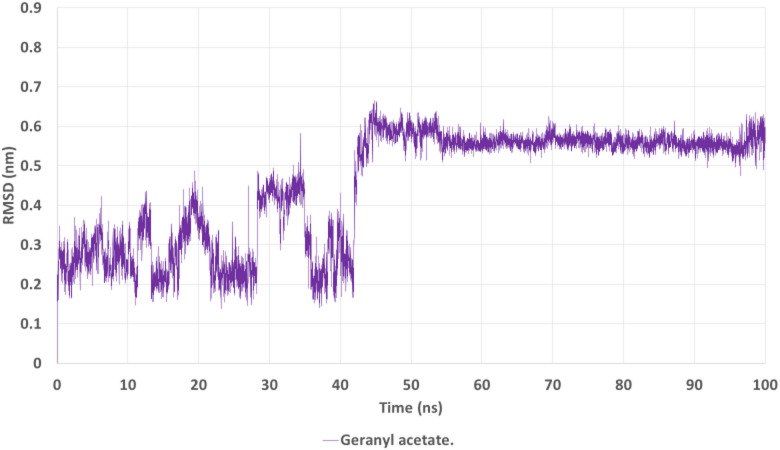
RMSD plot of the AChE–geranyl acetate complex over 100 ns.

The RMSD profile (Fig. 12) indicates an initial equilibration phase during the first ~ 40 ns, followed by a sharp increase and stabilization thereafter. The RMSD plateaued around ~ 0.55–0.6 nm, reflecting a stable protein–ligand conformation post-equilibration. The transition suggests that geranyl acetate **(18)** established a firm binding pose, and the protein-ligand complex maintained structural integrity for the remainder of the simulation.

The root-mean-square deviation (RMSD) indicates structural stabilization of the complex after ~ 40 ns, with equilibrium maintained around 0.55–0.6 nm, reflecting stable binding throughout the simulation.

**Fig. 13:**
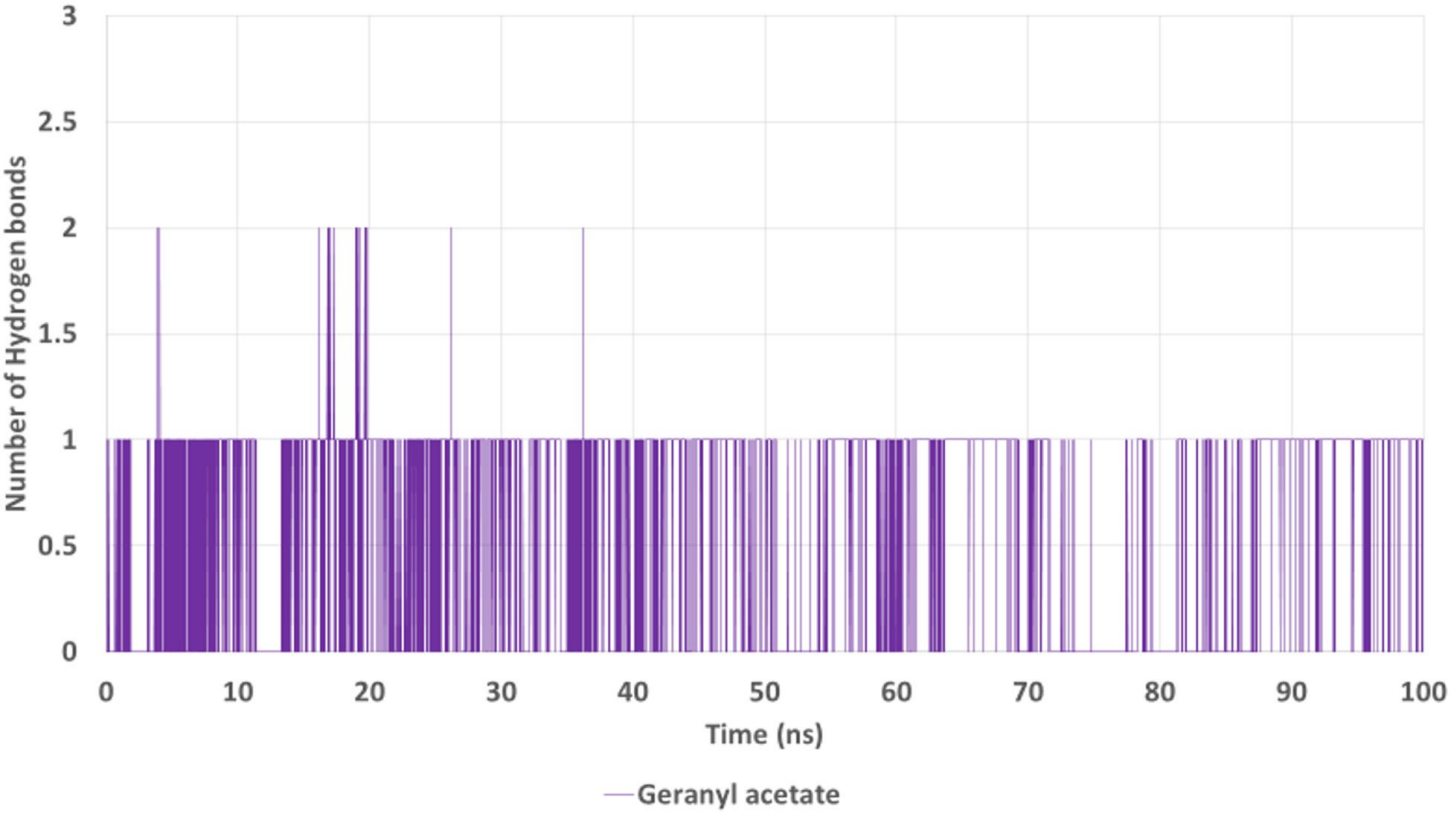
Hydrogen bond analysis of geranyl acetate with AChE over 100 ns.

The hydrogen bond analysis (Fig. 13) demonstrated that geranyl acetate formed intermittent but recurrent hydrogen bonds throughout the trajectory, primarily maintaining one conventional hydrogen bond at most time points. While the number of hydrogen bonds was modest, the consistent presence suggests a supportive role in stabilizing the ligand within the active site. This is in line with the docking interactions.

The number of hydrogen bonds fluctuates between 1 and 2, indicating transient but recurring interactions that contribute to the stability of the ligand within the active site.

**Fig. 14:**
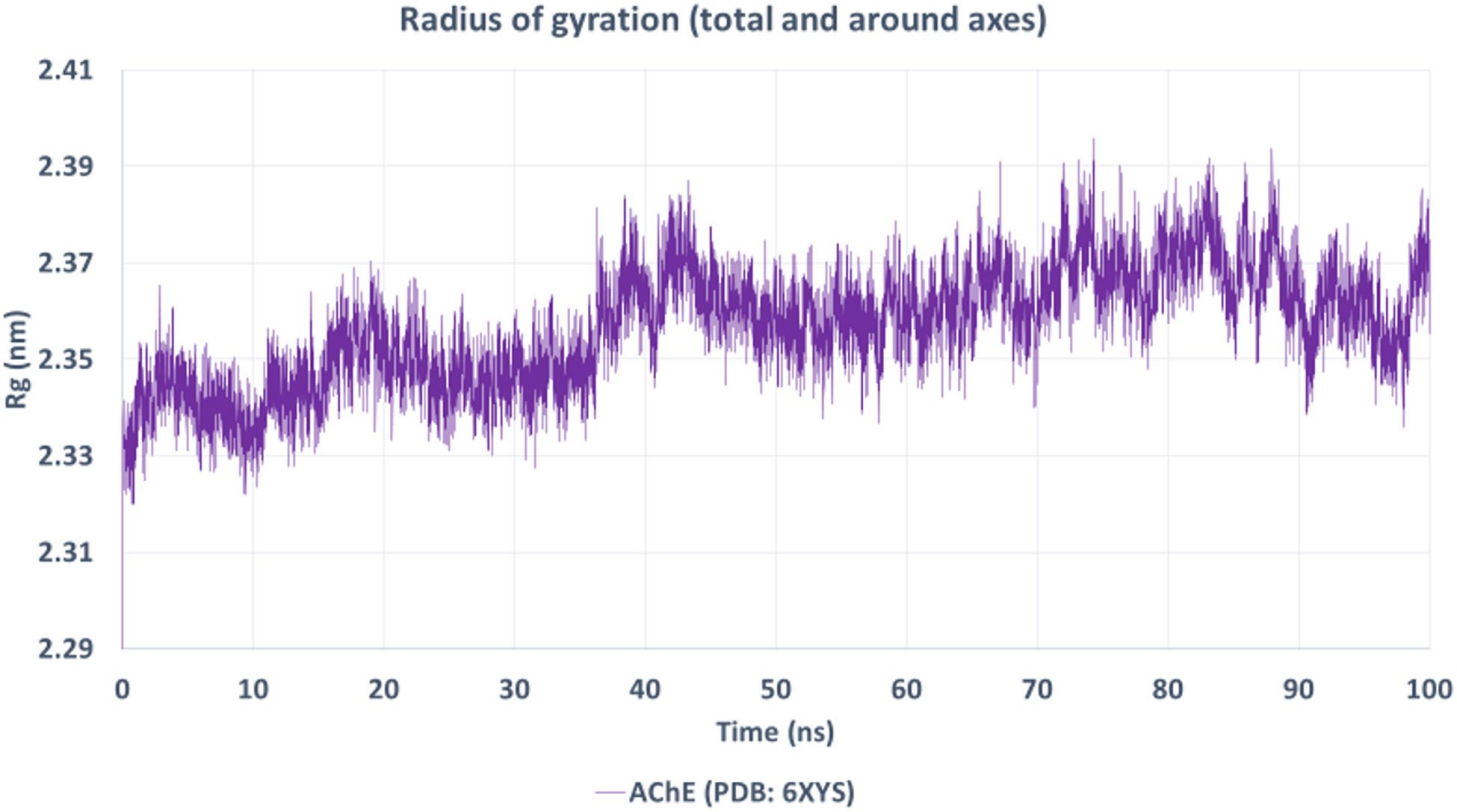
Radius of gyration (Rg) of the AChE–geranyl acetate complex.

The Rg plot (Fig. 14) reveals relatively stable values fluctuating between 2.33 and 2.39 nm, indicative of the compactness of the protein throughout the simulation. The absence of drastic changes suggests that geranyl acetate binding did not induce any significant unfolding or destabilization of the AChE protein structure.

The Rg remained stable between 2.33 and 2.39 nm throughout the simulation, suggesting that the protein maintained its compactness upon ligand binding.

**Fig. 15:**
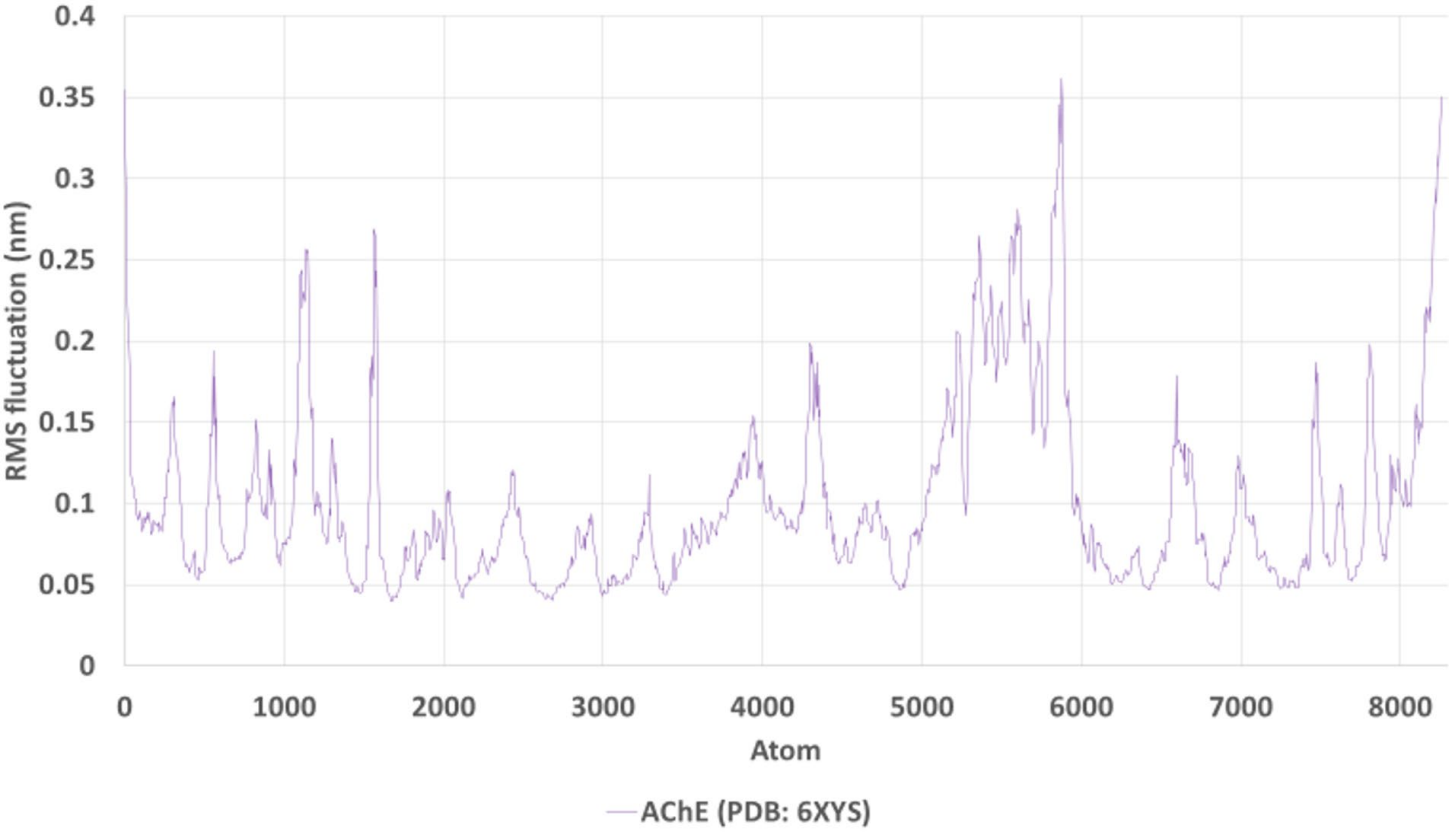
RMSF plot of AChE residues during 100 ns simulation.

The RMSF plot (Fig. 15) illustrates the per-residue flexibility of AChE. As expected, higher fluctuations were observed at the terminal regions, while the core catalytic residues and ligand-binding site showed limited mobility (< 0.2 nm). This low level of fluctuation in key residues further supports the stable binding conformation of geranyl acetate, preserving the structural rigidity of the active site.

The root-mean-square fluctuation (RMSF) reveals minimal flexibility in the ligand-binding region, with higher fluctuations observed in terminal and loop regions, confirming the structural stability of the binding site.

**Fig. 16:**
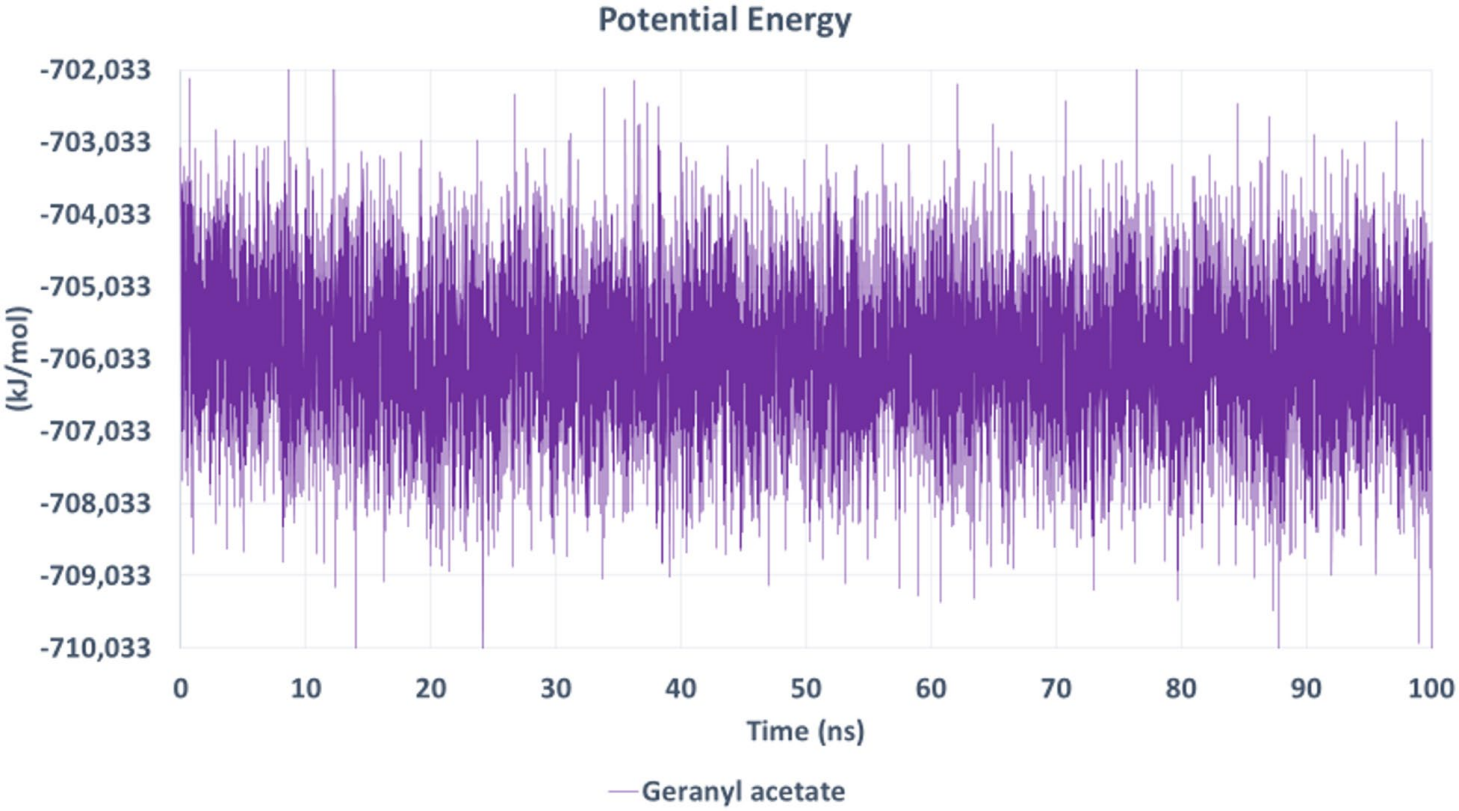
Potential energy profile of the AChE–geranyl acetate complex during simulation. The potential energy remained consistent throughout the 100 ns trajectory, indicating thermodynamic stability of the system under simulation conditions.

The total potential energy of the system (Fig. 16) remained relatively constant around − 705,000 kJ/mol, indicating system stability and the absence of large energy fluctuations or conformational collapses. The uniform energy distribution confirms that the simulated environment was energetically favorable for the AChE–geranyl acetate complex.

The MD simulation confirmed the docking results, highlighting geranyl acetate as a stable and favorable binder to AChE. The consistent RMSD, compact Rg, low active-site RMSF, persistent hydrogen bonding, and stable potential energy collectively suggest that geranyl acetate maintains a well-defined and energetically favorable interaction profile. These findings support its potential as an effective inhibitor of AChE activity and validate its selection for deeper structural and functional investigation. Given the central role of AChE in insect neurotransmission, the strong and stable interaction of geranyl acetate with this target supports its proposed insecticidal mechanism of action.

## Discussion

In this study, the oils were exposed to the heat of the distillation. This gives rise to a series of acid-catalyzed reactions, extensively studied and reported in the literature^38^. Mostly, the rearrangements of *α*- and *β*-pinene, *α*-phellandrene, sabinene, and *α*-thujene lead to the formation of *α*-terpineol, terpinen-4-ol, borneol, terpinolene, limonene, camphene, *γ*- and *α*-terpinene. Citral (neral and geranial) is believed to polymerize during the distillation process, whereas geranyl and neryl acetates seem to be the cause of *α*-terpineol increase, due to hydrolysis^39^.

Besides, some authors argued that the concentration of the oxygenated terpenes compounds is higher in the oils obtained by hydrodistillation compared to those obtained by the cold-press method^40,41^. Particularly in green fruits, a decrease in oxygenated monoterpenes and an increase in monoterpene hydrocarbons were observed during ripening.^42–44^.

In this study, *C. limon* essential oil is characterized by a high content of oxygenated monoterpenes (13.20% of the total oil) compared to *C. aurantium* and *C. margarita* oils (4.64% and 0.83%, respectively). The analysis of citrus essential oils revealed marked differences, particularly in terms of quantity, depending on the ripening stage of the fruit. In particular, the limonene content followed the order *C. aurantium* ≥ *C. margarita* > *C. limon*. Also, a marked difference was observed in the content of 2-thujene, *α*-pinene, (+)-sabinene, (+)-*β*-pinene, and α-myrcene. On the other hand, the sesquiterpene hydrocarbon essential oils content was in the following order: *C. margarita* > *C. limon > C. aurantium* (11.69, 9.68, 0.97%, respectively).

According to the literature, the chemical composition of essential oils varies depending on several factors, including the age of the plant, harvesting time, geographical location, environmental conditions, and methods of drying and extraction^45–50^. The Indian *C. limon* peel’s volatile oil differs from the Egyptian species, which contains **43** compounds, where limonene (55.40%) and neral (10.39%) were found as major compounds, followed by trans-verbenol (6.43%) and decanal (3.25%)^51^. The essential oil constituents reported in *C. limon* grown in Iran contained **11** compounds, with the main constituents, DL-limonene, *γ*-terpinene, 1-*β*-pinene, and 2-*β*-pinene, respectively^52,53^. The essential oil constituents reported in *C. limon* grown in Himachal Pradesh contained **9** compounds; *α*-terpineol was the main constituent (50.25%) in the peel oil^54^. Also, the essential oil constituents reported in *C. limon* grown in Benin contained limonene (70.4%), γ-terpinene (11.8%), and *β*-pinene (4.2%) as main constituents in peel oil^55^. Essential oil of Iraqi *C. limon* peels yielded **24** compounds, amounting to 97.28%. The major constituents of the essential oil of *C. limon* peels were limonene (29.52%), *β*-pinene (23.89%), citronellal (11.53%), and thymol (9.79%)^56^.

The Tunisian *C. aurantium* peel’s volatile oil differs from the Egyptian one, where limonene represents a percentage of 90.6%^57–60^. The essential oil constituents reported in *C. aurantium* grown in Eastern Morocco contained D-limonene as a major component^61^. Also, the Chott Mariem area of Sousse, and the Iranian *C. aurantium* peels, volatile oils contained D-limonene as the dominant^62,63^.

The French *C. margarita* peels volatile oil differently from the Egyptian, having **19** compounds, where monoterpene (96.51%) represents mainly limonene (93.10%), and myrcene (1.40%), while sesquiterpene (3.08%) represents mainly germacrene D (2.40%)^64^. The essential oil constituents reported in *C. margarita* grown in Taiwan and Algeria also contained limonene (84.71–95.06%), myrcene (1.88–3.52%), and germacrene D (0.93–4.70%), as majors^65–67^. The essential oil constituents reported in *C. margarita* grown in Greece contained **45** compounds, representing 99.7% of the total chromatographic area. Limonene was the principal component (93.8%). Other major compounds identified were myrcene (2.7%) and δ-germacrene (1.34%)^68^.

The literature review on essential oil components in *C. limon*,* C. aurantium*, and *C. margarita* cultivated in different regions corroborates some commonalities. Consequently, limonene, a hydrocarbon monoterpene, is invariably the most common ingredient in essential oils made from Citrus peels, making up typically between **30** and **96** percent of the oil. However, limonene can show lower levels, as in the Egyptian fruit. Also prevalent are the following substances: monoterpenes, which typically account for less than 25%, *β*-pinene, *α*-myrcene, (+)-sabinene, *γ-*terpinene, which can reach an abundance of 20%, 2–30%, 10%, and 20%, respectively.

Non-terpenoid or terpenoid compounds (phenols, aldehydes, ketones, and esters) are reported to be present (1–10%) or absent according to the cultivated region, but there are no commonalities among studies reporting these compounds to have an impact on the essential oil activity or not. Sesquiterpene hydrocarbons are the most varied group of all known chemicals. The most prevalent groupings also frequently include oxygenated monoterpene alcohols and monoterpene hydrocarbons.

Also, the insecticidal efficacy of peel oils from the three *Citrus* species, *Citrus limon*, *Citrus aurantium*, and *Citrus margarita*, against adult houseflies (*Musca domestica*) was evaluated. The findings revealed significant differences in toxicity among the tested oils. *C. limon* essential oil demonstrated the highest toxicity, with an LC_50_ value of 3.19 ppm and a 100% toxicity index. This high efficacy may be attributed to the specific composition of bioactive compounds present in *C. limon* oil, which could include higher concentrations of limonene and other potent monoterpenes known for their insecticidal properties. The steep slope (1.381) of the dose-response curve further indicates a strong and consistent toxic effect, suggesting that even small increases in concentration result in substantial increases in mortality.

*Citrus aurantium* essential oil also exhibited strong insecticidal activity, with an LC_50_ of 3.25 ppm and a toxicity index of 98.093%. Although its efficacy was nearly equivalent to that of *C. limon*, the lower slope (0.802) suggests a less pronounced increase in mortality with increasing concentrations. This difference in slope may reflect variations in the oil’s chemical profile or the presence of synergistic or antagonistic compounds.

In contrast, *C. margarita* oil was markedly less effective, with an LC_50_ of 479.12 ppm and a toxicity index of only 0.666%. The much higher LC_50_ value indicates that significantly greater concentrations are required to achieve the same level of mortality as the other oils. This suggests that *C. margarita* oil either lacks sufficient quantities of active insecticidal constituents or contains compounds that reduce its overall toxicity.

These results highlight the potential of *C. limon* and *C. aurantium* peel oils as effective botanical insecticides for the management of housefly populations. Their high efficacy at low concentrations makes them promising alternatives to synthetic chemical insecticides, which are often associated with resistance development and environmental concerns. The poor performance of *C. margarita* oil, however, suggests that not all citrus oils are equally effective, emphasizing the importance of species selection in developing plant-based pest control agents.

Further research should focus on identifying the specific active components responsible for the observed toxicity, as well as evaluating the safety and practicality of using these oils in real-world settings. Additionally, studies on the mode of action and potential for resistance development would provide valuable insights for integrated pest management strategies.^69^.

Based on our experimental findings, we compiled a dataset of key protein targets involved in insecticide resistance mechanisms in *Musca domestica*. These targets were selected based on their critical roles in neurotransmission, detoxification, and metabolic regulation, as well as their relevance in overcoming pesticide resistance. The dataset includes proteins such as acetylcholinesterase (AChE), GABA receptors, cytochrome P450 enzymes, and glutathione S-transferases, which have been linked to resistance to insecticides. The protein targets were curated from publicly available databases such as GenBank and further validated through our network pharmacology analysis. To further investigate the interactions between the bioactive compounds identified in the citrus oils and the insecticide resistance targets (Table S1)^70^. The STITCH database was used to construct a protein–compound interaction network.

The dataset compiled in this study highlights the central role of several protein families in insecticide resistance in *Musca domestica*. Acetylcholinesterase (AChE) and GABA receptors are crucial for neurotransmission, while cytochrome P450 enzymes and glutathione S-transferases (GSTs) play a central role in detoxification and metabolic regulation. Their inclusion reflects the multifaceted nature of resistance, which involves both target-site insensitivity and enhanced metabolic detoxification. The validation of these targets through network pharmacology analysis strengthens the reliability of the dataset and provides a robust foundation for exploring novel bioactive modulators.

Citrus-derived compounds, specifically D-limonene, α-pinene, geranyl acetate, and terpinenol, interact significantly with resistance-related proteins, according to the STITCH-based protein–compound interaction network. These findings suggest that naturally occurring chemicals derived from plants may influence pesticide resistance pathways by modifying neurotransmission and detoxification processes. D-limonene and α-pinene, for instance, are known to have neuroactive qualities; their interactions with GABA and AChE receptors point to possible disruption of synaptic communication. The binding of these substances to P450 enzymes and cytochrome P450 regulation suggests potential detoxification interference, which lowers the metabolic clearance of insecticides.

A deeper understanding of the intricacy of these interactions was made possible by the Cytoscape display. Geranyl acetate emerged as a central node, forming strong connections with multiple protein targets. This suggests that geranyl acetate may play a pivotal role in disrupting neurotransmission and enhancing insecticide susceptibility. Network topology underscores the potential of certain bioactive compounds to act as synergists, weakening resistance mechanisms by targeting multiple pathways simultaneously.

These findings have important implications for integrated pest management (IPM). The ability of citrus oil compounds to interact with resistance-associated proteins suggests their potential as natural synergists or alternative agents to conventional insecticides. By modulating key resistance pathways, these compounds could restore insecticide efficacy or reduce reliance on synthetic chemicals. Moreover, the use of natural products aligns with sustainable pest control strategies, minimizing environmental and health risks.

While the interaction networks provide valuable mechanistic insights, further experimental validation is required to confirm the functional outcomes of these compound–protein interactions. Electrophysiological assays, enzyme inhibition studies, and in vivo bioassays would help establish the extent to which citrus oil metabolites can overcome resistance. Additionally, exploring the structural basis of these interactions through molecular docking and dynamics simulations could refine our understanding of their binding specificity and potency.

## Conclusion

This study investigated the constituents of the essential oils from three *Citrus* species, *C. limon*, *C. aurantium*, and *C. margarita*, utilizing GC/MS analysis. The identification of **31** compounds revealed that monoterpene hydrocarbons were the predominant class, comprising between 98.71% and 85.99% of the total isolated compounds. Furthermore, we evaluated the insecticidal efficacy of the three species against the housefly, *Musca domestica*, and determined that *C. limon* oil, with an LC_50_ of 3.19 ppm, exhibited greater effectiveness against adult houseflies than *C. aurantium* and *C. margarita* oils. *C. limon* oil exhibits a toxicity level of 100%, but *C. aurantium* demonstrates comparable efficacy with an LC_50_ of 3.25 ppm and a toxicity index of 98.093%. Conversely, *C. margarita* exhibited the lowest toxicity index at 0.666%, in contrast to *C. limon* and *C. aurantium*.

A comprehensive in silico analysis identified geranyl acetate **18** as a strong candidate for insecticidal activity through AChE inhibition. Functional enrichment and network analysis revealed its involvement in pathways critical to insect survival and resistance, while docking and MD simulations confirmed its stable and high-affinity interaction with AChE. By targeting the neural and detoxification systems of *Musca domestica*, geranyl acetate **18** demonstrates the potential to act as an effective natural insecticide. These results provide a molecular foundation for future experimental validation and the development of eco-friendly pest control agents based on citrus.

## Supplementary Information

Below is the link to the electronic supplementary material.

Supplementary Material 1

## Data Availability

All data generated or analyzed during this study are included in this published article (and its Supplementary Information files).
