# Supplementary material for: Chemical composition and insecticidal activity of essential oils from Citrus limon, Citrus aurantium, and Citrus margarita against Musca domestica
Source: Sci Rep. 2026 Apr 20;16:12858. doi: 10.1038/s41598-026-48049-6 (PMC13096227; doi:10.1038/s41598-026-48049-6)
Supplement: Supplementary file 1 — Supplementary Material 1 [file 41598_2026_48049_MOESM1_ESM.docx]

**Chemical Composition and Insecticidal Activity of Essential Oils from *Citrus limon*, Citrus *aurantium*, and *Citrus margarita* Against *Musca domestica***

Abeer H. Elmaidomy^1,^ Esraa M. Mohamed^2^, Radwa Taher Mohie el-dien^3^, Hesham A. Abou-Zied^4^, Azza A. Mostafa^5^, Riham H. Taha^5^, Mohamed A. El-Badry^5^, Usama Ramadan Abdelmohsen^3,6*^, Khayrya A. Youssif^7^.

^1^Department of Pharmacognosy, Faculty of Pharmacy, Beni-Suef University, Beni-Suef 62514, Egypt. [Abeer011150@pharm.bsu.edu.eg](mailto:Abeer011150@pharm.bsu.edu.eg)

^2^Department of Pharmacognosy, Faculty of Pharmacy, MUST, Giza, 12566, Egypt.

^3^Department of Pharmacognosy, Faculty of Pharmacy, Deraya University, Minia 61111, Egypt.

^4^Department of Medicinal Chemistry, Faculty of Pharmacy, Deraya University, Minia 61111, Egypt. [hisham.alaa@deraya.edu.eg](mailto:hisham.alaa@deraya.edu.eg)

^5^Research Institute of Medical Entomology (RIME), General Organization for Teaching Hospitals and Institutes (GOTHI), Giza, Egypt.

^6^Department of Pharmacognosy, Faculty of Pharmacy, Minia University, 61519 Minia, Egypt.

^7^Department of Pharmacognosy, Faculty of Pharmacy, El Saleheya El Gadida University, El Sharqya, Egypt. [Khayrya.youssif@gmail.com](mailto:Khayrya.youssif@gmail.com).

* **Correspondence author:**

usama.ramadan@mu.edu.eg (U.R.A.)

**Abstract**

There are numerous culinary and therapeutic applications for citrus peel essential oils. In this study, we used GC/MS analysis to investigate the differences in yield and chemical composition of the essential oils extracted from the fresh peels of three *Citrus* species: *C. limon, C.* *aurantium*, and *C. margarita*. In total, 21 compounds were isolated from *C. limon*, 15 from *C. aurantium*, and 9 from *C. margarita*. The most prevalent constituents were α-myrcene (ranging from 7.05 to 12.80% and 11.50%) and D-limonene (ranging from 15.46 to 71.71% and 71.59%).

Furthermore, the essential oils of the three citrus species were tested for their ability to kill adult houseflies (*Musca domestica*). The results showed that *C. limon* oil was more effective than *C. aurantium* and *C. margarita* oils, with an LC_50_ value of 3.19 and a 100% toxicity index. An *in silico* study assessed 31 Citrus-derived compounds for their insecticidal potential against *Musca domestica* by targeting acetylcholinesterase (AChE). Network and docking analysis identified geranyl acetate as the top binder (−10.2 kcal/mol), interacting with key residues involved in neurotransmission. A 100 ns molecular dynamics confirmed its stable binding and supports its potential role as a natural AChE inhibitor for insect control. These findings offer a molecular basis for upcoming experimental verification and advancement of environmentally benign pesticides derived from citrus.

**Keywords:** Citrus species, GC/MS analysis, essential oils, insecticidal activity, *Musca domestica.*

**Table S1.** List of proteins associated with insecticide resistance and neural or metabolic functions in *Musca domestica*.

| Protein | Online library Source |
| --- | --- |
| A4_EXTRA domain-containing protein | GenBank |
| Aminopeptidase | GenBank |
| Biopterin-dependent aromatic amino acid hydroxylase | GenBank |
| Acetylcholinesterase-like; type-B carboxylesterase/lipase family | GenBank |
| Carboxylic ester hydrolase; type-B carboxylesterase/lipase family | GenBank |
| Nicotinic acetylcholine receptor α subunit (nAChR-α) | GenBank |
| Nicotinic acetylcholine receptor β subunit (nAChR-β) | GenBank |
| Carn_acyltransferase domain-containing protein | GenBank |
| Voltage-gated sodium channel (VGSC, para gene) | GenBank |
| Octopamine receptor (Octβ2R) | GenBank |
| Catalase | GenBank |
| Catalase domain-containing protein | GenBank |
| Chitin binding protein | GenBank |
| Choline/ethanolamine kinase | GenBank |
| COesterase domain-containing protein | GenBank |
| COesterase domain-containing protein | GenBank |
| DNA ligase | GenBank |
| EF-hand domain-containing protein | GenBank |
| GABA receptor subunit beta; ligand-gated chloride channel (TC 1.A.9.5) | GenBank |
| Glutathione peroxidase | GenBank |
| MFS domain-containing protein | GenBank |
| Voltage-gated calcium channel (Cav subunit α1) | GenBank |
| RING-type domain-containing protein | GenBank |
| S-(hydroxymethyl)glutathione dehydrogenase; Class-III ADH | GenBank |
| Signal recognition particle subunit SRP72 | GenBank |
| Superoxide dismutase [Cu-Zn] | GenBank |
| Heat shock protein 90 (Hsp90) | GenBank |
| ATP-binding cassette transporter (ABC-G subfamily) | GenBank |
| Glutamate-gated chloride channel (GluCl) | GenBank |
| Histamine-gated chloride channel (HisCl1) | GenBank |
| Octopamine receptor (Octβ2R) | GenBank |
| Cytochrome P450 family | GenBank |
| Ligand-gated ion channel (TC 1.A.9) family | GenBank |
| Ligand-gated ion channel (TC 1.A.9) family | GenBank |
| protein kinase superfamily (Tyr kinase family) | GenBank |
| Sodium: Solute Symporter (SSF, TC 2.A.21) family | GenBank |

**Table S2.** Binding energies (kcal/mol) and RMSD values of the **31** bioactive compounds from *Citrus* species docked into the active pocket of acetylcholinesterase (AChE) from *Musca domestica* (modeled using *Drosophila melanogaster* homolog, PDB ID: 6XYS).

| Compound No. | Compound | S score (kcal/mol) | RMSD (Å) |
| --- | --- | --- | --- |
| **1** | 2-Thujene | -7.12 | 0.81 |
| **2** | Alpha-Pinene | -8.85 | 0.62 |
| **3** | Camphene | -8.2 | 2.21 |
| **4** | (+)-Sabinene | -7.8 | 2.24 |
| **5** | (+)-Beta-Pinene | -6.47 | 1.96 |
| **6** | Alpha-Myrcene | -6.47 | 1.05 |
| **7** | Alpha-Terpinene | -6.17 | 0.68 |
| **8** | o-Cymene | -8.6 | 1.73 |
| **9** | D-Limonene | -9.5 | 0.78 |
| **10** | Alpha-Ocimene | -8.12 | 0.72 |
| **11** | Gamma-Terpinene | -6.06 | 1.39 |
| **12** | p-Mentha-1,4(8)-diene | -8.91 | 0.56 |
| **13** | 3,7-Dimethylocta-1,6-dien-3-ol | -8.5 | 2.14 |
| **14** | Terpinen-4-ol | -9.2 | 0.89 |
| **15** | Alpha-Terpineol | -6.55 | 1.69 |
| **16** | Alpha-Citral (Geranial) | -6.55 | 1.06 |
| **17** | Neryl acetate | -6.91 | 1.44 |
| **18** | Geranyl acetate | -10.2 | 0.62 |
| **19** | Caryophyllene | -7.3 | 0.83 |
| **20** | cis-Alpha-Bergamotene | -6.87 | 2.25 |
| **21** | cis-Alpha-Bisabolene | -7.84 | 1.9 |
| **22** | Linalool | -6.42 | 2.19 |
| **23** | cis-Linalool oxide | -6.88 | 2.11 |
| **24** | Linalyl acetate | -7.1 | 1.58 |
| **25** | (−)-(1S,2R,4R)-Beta-Fenchol | -7.37 | 2.16 |
| **26** | Decanal | -8.36 | 0.66 |
| **27** | Germacrene D | -6.6 | 0.85 |
| **28** | Nerolidol | -7.54 | 0.58 |
| **29** | Lepidozene | -7.78 | 1.09 |
| **30** | Germacrene B | -6.14 | 1.2 |
| **31** | 3-Isopropenyl-1-isopropyl-4-methyl-4-vinyl-1-cyclohexene | -7.82 | 0.99 |

**Table S3**. Insecticidal activity of *C. limon* essential oils against *Musca domestica*

| *Citrus limon*  concentrations (%) | Replicates | Dead | Alive | Total | Mortality percentage (%) after 2hrs  exposure | | | | | LC_50_ (%) | LC_90_ (%) | Slope |
| --- | --- | --- | --- | --- | --- | --- | --- | --- | --- | --- | --- | --- |
| 1 | R1 | 6 | 19 | 25 | 24 | | | | 3.19 | | 27.05 | 1.38 |
|  | R2 | 7 | 18 | 25 | 28 | | |  | | |  |  |
|  | R3 | 6 | 19 | 25 | 24 | | |  |  |  |  |  |
|  | R4 | 6 | 19 | 25 | 24 | | |  |  |  |  |  |
|  | Mean | 6.25 |  |  | 25 | | |  |  |  |  |  |
| 5 | R1 | 14 | 11 | 25 | 56 | |  | | | |  |  |
|  | R2 | 15 | 10 | 25 | 60 | |  |  |  |  |  |  |
|  | R3 | 16 | 9 | 25 | 64 | |  |  |  |  |  |  |
|  | R4 | 15 | 10 | 25 | 60 | |  |  |  |  |  |  |
|  | Mean | 15 |  |  | 60 | |  |  |  |  |  |  |
| 10 | R1 | 17 | 8 | 25 | 68 |  | | | | |  |  |
|  | R2 | 18 | 7 | 25 | 72 |  |  |  |  |  |  |  |
|  | R3 | 19 | 6 | 25 | 76 |  |  |  |  |  |  |  |
|  | R4 | 18 | 7 | 25 | 72 |  |  |  |  |  |  |  |
|  | Mean | 18 |  |  | 72.5 |  |  |  |  |  |  |  |
| 15 | R1 | 21 | 4 | 25 | 84 |  |  |  |  |  |  |  |
|  | R2 | 22 | 3 | 25 | 88 |  |  |  |  |  |  |  |
|  | R3 | 21 | 4 | 25 | 84 |  |  |  |  |  |  |  |
|  | R4 | 21 | 4 | 25 | 84 |  |  |  |  |  |  |  |
|  | Mean | 21.25 |  |  | 85 |  |  |  |  |  |  |  |

**Table S4**. Insecticidal activity of *C. aurantium* essential oils against *Musca domestica*

| *Citrus aurantium*  concentrations (%) | Replicates | Dead | Alive | Total | Mortality percentage (%) after 2hrs  exposure | LC_50_ (%) | LC_90_ (%) | Slope |
| --- | --- | --- | --- | --- | --- | --- | --- | --- |
| 0.5 | R1 | 4 | 21 | 25 | 16 | 3.25 | 129.0 | 0.8018 |
|  | R2 | 5 | 20 | 25 | 20 |  |  |  |
|  | R3 | 6 | 19 | 25 | 24 |  |  |  |
|  | R4 | 5 | 20 | 25 | 20 |  |  |  |
|  | Mean | 5 |  |  | 20 |  |  |  |
| 1 | R1 | 9 | 16 | 25 | 36 |  |  |  |
|  | R2 | 10 | 15 | 25 | 40 |  |  |  |
|  | R3 | 11 | 14 | 25 | 44 |  |  |  |
|  | R4 | 10 | 15 | 25 | 40 |  |  |  |
|  | Mean | 10 |  |  | 40 |  |  |  |
| 5 | R1 | 14 | 11 | 25 | 56 |  |  |  |
|  | R2 | 15 | 10 | 25 | 60 |  |  |  |
|  | R3 | 16 | 9 | 25 | 64 |  |  |  |
|  | R4 | 15 | 10 | 25 | 60 |  |  |  |
|  | Mean | 15 |  |  | 60 |  |  |  |
| 15 | R1 | 16 | 9 | 25 | 64 |  |  |  |
|  | R2 | 17 | 8 | 25 | 68 |  |  |  |
|  | R3 | 16 | 9 | 25 | 64 |  |  |  |
|  | R4 | 16 | 9 | 25 | 64 |  |  |  |
|  | Mean | 16.25 |  |  | 65 |  |  |  |
| 20 | R1 | 18 | 7 | 25 | 72 |  |  |  |
|  | R2 | 19 | 6 | 25 | 76 |  |  |  |
|  | R3 | 19 | 6 | 25 | 76 |  |  |  |
|  | R4 | 19 | 6 | 25 | 76 |  |  |  |
|  | Mean | 18.75 |  |  | 75 |  |  |  |

**Table S5**. Insecticidal activity of *C. margarita* essential oils against *Musca domestica*

| *Citrus margarita* concentrations (%) | Replicates | Dead | Alive | Total | Mortality percentage (%) after 2hrs  exposure | LC_50_ (%) | LC_90_ (%) | Slope |
| --- | --- | --- | --- | --- | --- | --- | --- | --- |
| 1 | R1 | 1 | 24 | 25 | 4 | 479.11 | 5486.5 | 0.6225 |
|  | R2 | 1 | 24 | 25 | 4 |  |  |  |
|  | R3 | 2 | 23 | 25 | 8 |  |  |  |
|  | R4 | 1 | 24 | 25 | 4 |  |  |  |
|  | Mean | 1.25 |  |  | 5 |  |  |  |
| 5 | R1 | 2 | 23 | 25 | 8 |  |  |  |
|  | R2 | 3 | 22 | 25 | 12 |  |  |  |
|  | R3 | 3 | 22 | 25 | 12 |  |  |  |
|  | R4 | 2 | 23 | 25 | 8 |  |  |  |
|  | Mean | 2.5 |  |  | 10 |  |  |  |
| 10 | R1 | 3 | 22 | 25 | 12 |  |  |  |
|  | R2 | 4 | 21 | 25 | 16 |  |  |  |
|  | R3 | 4 | 21 | 25 | 16 |  |  |  |
|  | R4 | 4 | 21 | 25 | 16 |  |  |  |
|  | Mean | 3.75 |  |  | 15 |  |  |  |
| 15 | R1 | 4 | 21 | 25 | 16 |  |  |  |
|  | R2 | 4 | 21 | 25 | 16 |  |  |  |
|  | R3 | 5 | 20 | 25 | 20 |  |  |  |
|  | R4 | 4 | 21 | 25 | 16 |  |  |  |
|  | Mean | 4.25 |  |  | 17.5 |  |  |  |
| 20 | R1 | 5 | 20 | 25 | 20 |  |  |  |
|  | R2 | 5 | 20 | 25 | 20 |  |  |  |
|  | R3 | 4 | 21 | 25 | 16 |  |  |  |
|  | R4 | 6 | 19 | 25 | 24 |  |  |  |
|  | Mean | 5 |  |  | 20 |  |  |  |
| 30 | R1 | 5 | 20 | 25 | 20 |  |  |  |
|  | R2 | 6 | 19 | 25 | 24 |  |  |  |
|  | R3 | 6 | 19 | 25 | 24 |  |  |  |
|  | R4 | 5 | 20 | 25 | 20 |  |  |  |
|  | Mean | 5.5 |  |  | 22.5 |  |  |  |
| 40 | R1 | 6 | 19 | 25 | 24 |  |  |  |
|  | R2 | 7 | 18 | 25 | 28 |  |  |  |
|  | R3 | 6 | 19 | 25 | 24 |  |  |  |
|  | R4 | 6 | 19 | 25 | 24 |  |  |  |
|  | Mean | 6.25 |  |  | 25 |  |  |  |
